# Macrophage plasticity: signaling pathways, tissue repair, and regeneration

**DOI:** 10.1002/mco2.658

**Published:** 2024-08-01

**Authors:** Lingfeng Yan, Jue Wang, Xin Cai, Yih‐Cherng Liou, Han‐Ming Shen, Jianlei Hao, Canhua Huang, Gaoxing Luo, Weifeng He

**Affiliations:** ^1^ Institute of Burn Research State Key Laboratory of Trauma and Chemical Poisoning the First Affiliated Hospital of Army Medical University (the Third Military Medical University) Chongqing China; ^2^ Chongqing Key Laboratory for Wound Damage Repair and Regeneration Chongqing China; ^3^ Department of Biological Sciences Faculty of Science National University of Singapore Singapore Singapore; ^4^ National University of Singapore (NUS) Graduate School for Integrative Sciences and Engineering National University of Singapore Singapore Singapore; ^5^ Faculty of Health Sciences University of Macau Macau China; ^6^ Guangdong Provincial Key Laboratory of Tumor Interventional Diagnosis and Treatment Zhuhai Institute of Translational Medicine Zhuhai People's Hospital (Zhuhai Clinical Medical College of Jinan University) Jinan University Zhuhai Guangdong China; ^7^ The Biomedical Translational Research Institute Faculty of Medical Science Jinan University Guangzhou Guangdong China; ^8^ State Key Laboratory of Biotherapy and Cancer Center West China Hospital and West China School of Basic Medical Sciences and Forensic Medicine Sichuan University, and Collaborative Innovation Center for Biotherapy Chengdu China

**Keywords:** epigenetic regulation, macrophages, plasticity, signaling pathways, tissue repair

## Abstract

Macrophages are versatile immune cells with remarkable plasticity, enabling them to adapt to diverse tissue microenvironments and perform various functions. Traditionally categorized into classically activated (M1) and alternatively activated (M2) phenotypes, recent advances have revealed a spectrum of macrophage activation states that extend beyond this dichotomy. The complex interplay of signaling pathways, transcriptional regulators, and epigenetic modifications orchestrates macrophage polarization, allowing them to respond to various stimuli dynamically. Here, we provide a comprehensive overview of the signaling cascades governing macrophage plasticity, focusing on the roles of Toll‐like receptors, signal transducer and activator of transcription proteins, nuclear receptors, and microRNAs. We also discuss the emerging concepts of macrophage metabolic reprogramming and trained immunity, contributing to their functional adaptability. Macrophage plasticity plays a pivotal role in tissue repair and regeneration, with macrophages coordinating inflammation, angiogenesis, and matrix remodeling to restore tissue homeostasis. By harnessing the potential of macrophage plasticity, novel therapeutic strategies targeting macrophage polarization could be developed for various diseases, including chronic wounds, fibrotic disorders, and inflammatory conditions. Ultimately, a deeper understanding of the molecular mechanisms underpinning macrophage plasticity will pave the way for innovative regenerative medicine and tissue engineering approaches.

## Graphical abstract

The rapid and orderly process of wound healing depends on the normal function of M1 and M2 and timely phenotypic transformation. However, when M1 phenotype is overexpressed or phenotypic transformation is delayed, it often leads to the pathological healing process of the wound.

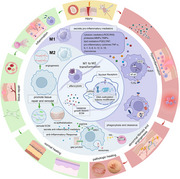

## 1 INTRODUCTION

Macrophages, the versatile sentinels of the innate immune system, play critical roles in host defense, tissue homeostasis, and disease pathogenesis. Macrophages exhibit extraordinary plasticity, enabling them to adapt to diverse tissue microenvironments and perform various functions. This plasticity is exemplified by the ability of macrophages to polarize into distinct functional states in response to various stimuli, a process orchestrated by complex signaling pathways and transcriptional networks. In the past decade, there has been a surge of interest in elucidating the molecular mechanisms governing macrophage plasticity and their implications for tissue repair and regeneration. Macrophages play pivotal roles in all stages of the tissue repair process, from the initial inflammatory response to the resolution of inflammation and tissue remodeling.^1^, ^2^, ^3^ Dysregulation of macrophage function can lead to impaired wound healing, fibrosis, and chronic inflammation, underscoring the importance of understanding the signaling pathways that control macrophage activation states. Recent studies have shed light on the complex network of transcription factors, epigenetic regulators, and metabolic pathways that shape the macrophage transcriptional landscape and functional properties.^4^, ^5^, ^6^, ^7^, ^8^

At present, the classification of macrophages into M1 and M2 types has been widely adopted in scientific research.^9^, ^10^, ^11^, ^12^, ^13^, ^14^, ^15^, ^16^ Through this extreme classification, macrophages are divided according to the functional differences of proinflammatory bactericidal and anti‐inflammatory and anti‐inflammatory prorepair.^17^, ^18^, ^19^ While this dichotomy provided a valuable framework for understanding macrophage heterogeneity, recent advances in single‐cell technologies and systems biology approaches have revealed a spectrum of activation states that extend beyond the M1/M2 paradigm.^20^, ^21^ Therefore, further research is necessary to reconcile these findings and provide a more comprehensive understanding of macrophage phenotypes and functions. Despite this ongoing debate, the M1 and M2 classification continues to describe the different polarization states of macrophages in the study of tissue healing. During tissue repair, M1 and M2 macrophages fulfill distinct functions, and the timely transition of macrophages from an M1 to an M2 phenotype plays a critical role in wound healing and tissue regeneration.^22^

Through this review, we aim to clarify the key pathways and epigenetic regulation that regulate the polarization of macrophages and discuss their roles in the repair and regeneration of various tissue damage to provide ideas and approaches for clinically diagnosing and treating macrophages as targets. The review is structured into four main sections. In the first section, we provide an overview of the historical perspective on macrophage plasticity and the evolution of the M1/M2 paradigm. We then delve into the spectrum of macrophage activation states revealed by recent single‐cell studies and discuss the limitations of the traditional classification system. The second section focuses on the signaling pathways that control macrophage polarization, emphasizing the roles of TLRs, signal transducer and activator of transcription (STAT) proteins, nuclear receptors, and microRNAs (miRNAs). We also discuss the emerging concepts of macrophage metabolic reprogramming and epigenetic regulation, highlighting their importance in shaping macrophage functional properties. The third section explores the functional significance of macrophage plasticity in tissue repair and regeneration, providing examples from cutaneous wound healing, skeletal muscle regeneration, and liver repair. We discuss macrophages’ dynamic roles in the repair process's different stages and the consequences of macrophage dysfunction in pathological conditions. The final section addresses the therapeutic potential of targeting macrophage polarization pathways, discussing the current strategies and future directions for modulating macrophage function in chronic wounds, fibrotic diseases, and inflammatory disorders.

## 2 MACROPHAGE PLASTICITY: FROM HISTORICAL ORIGINS TO CONTEMPORARY INSIGHTS

Macrophages, the versatile sentinels of the immune system, exhibit remarkable plasticity that enables them to adapt to diverse tissue microenvironments and perform a wide array of functions.^23^ First discovered by Élie Metchnikoff in 1882, these phagocytic cells were recognized for their roles in immunity and inflammation.^24^, ^25^ Over the past century, our understanding of macrophage heterogeneity and plasticity has dramatically expanded, revealing their critical involvement in tissue homeostasis, wound healing, and disease pathogenesis. The traditional M1/M2 classification system, proposed by Mills et al.^15^ in 2000, provided a valuable framework for understanding macrophage polarization. M1 macrophages, activated by interferon‐γ (IFN‐γ) and lipopolysaccharide (LPS), exhibit proinflammatory properties and potent microbicidal activity. In contrast, M2 macrophages, induced by interleukin‐4 (IL‐4) and IL‐13, display anti‐inflammatory and tissue‐repair functions. While this dichotomy captures the extremes of macrophage activation, recent advances have revealed a spectrum of activation states that extend beyond the M1/M2 paradigm. This section discusses the historical origins of macrophage plasticity research and highlights the latest discoveries that have reshaped our understanding of this phenomenon. We explore the molecular mechanisms underlying macrophage plasticity, including transcriptional regulation, epigenetic modifications, and metabolic reprogramming. Furthermore, we discuss the functional significance of macrophage plasticity in health and disease, focusing on tissue‐specific adaptations and the role of trained immunity. Finally, we outline this rapidly evolving field's challenges and future directions.

### 2.1 Historical perspective on macrophage plasticity

Macrophage plasticity emerged in the 1960s when Mackaness et al.^26^, ^27^, ^28^, ^29^, ^30^, ^31^ reported two distinct macrophage activation states responding to cytokines. Type 1 macrophages, now known as M1, exhibited enhanced microbicidal activity against intracellular pathogens like Mycobacterium tuberculosis. Type 2 macrophages, now called M2, dampened inflammation and promoted extracellular matrix remodeling. In the 1980 and 1990s, the phenotypic and functional differences between M1 and M2 macrophages came into sharper focus. Stein et al.^32^ found that M1 macrophages produced high levels of proinflammatory cytokines, such as tumor necrosis factor‐α (TNF‐α), IL‐1, IL‐6, and IL‐12. This enabled them to stimulate T‐cell responses and potently unleash oxidative attacks against pathogens. Conversely, M2 macrophages secreted anti‐inflammatory cytokines like IL‐10 and expressed high arginase‐1 (ARG‐1) levels, allowing them to suppress immune responses and promote tissue repair.^32^ Based on arginine metabolism, the M1/M2 classification system was consolidated by Mills et al.^15^ in 2000, drawing parallels with T helper 1 (Th1) and Th2 lymphocyte polarization. While this dichotomy provided a helpful framework, it oversimplified the complex spectrum of macrophage activation states observed in vivo (Table 1).

**TABLE 1:** History of macrophage polarization studies.

Time	Finder	Conclusion	References
1863	Recklinghausen	Mononuclear amoeba cells were discovered and called connective tissue bodies, distinguishing them from pus bodies.	^33^
1882	Metchnikoff	He discovered a cell capable of engulfing particles and fragments of carmine dye. He named the cells macrophages and the process of phagocytosis.	^24^, ^34^
1898	Kupffer & Browicz	Kupffer discovered the star‐shaped cells in the liver, and Browicz identified the star‐shaped cells as distinct macrophages of the liver.	^35^
1924	Aschoff	He classified the cells in the body capable of swallowing dyes as the reticuloendothelial system.	^36^
1967	Mackaness	He discovered that macrophages could attack bacteria indiscriminately after infection and defined “macrophage activation” for the first time.	^26^, ^31^
1968	van Furth & Cohn	The mononuclear macrophage system is defined as a population whose life history is defined: promonocytes in the bone marrow, monocytes in the blood, and macrophages in the tissue.	^37^, ^38^
1992	Stein	He was the first to discover that IL‐4‐activated M2 macrophages were distinct from the classical activation of macrophages.	^32^
2000	Mills	He further clarified the M1/M2 classification system of macrophages based on arginine metabolism.	^15^
2012	Quintin	He was the first to propose functional reprogramming of monocytes	^39^
2014	Lavin	He determined that macrophages from different tissues have tissue‐specific enhancer landscapes, highlighting the importance of the microenvironment for the macrophage regulatory landscape.	^40^
2014	Xue	He used the scRNA‐seq technique to identify 49 distinct subpopulations of macrophages through 28 different stimuli.	^41^
2014	Cheng	He proposed that training immunity relies on the aerobic glycolysis pathway induced by the Akt–mTOR–HIF‐1α pathway.	^42^
2016	Arts	He proposed an essential role for metabolic regulation in the functional reprogramming of macrophages and discovered that the transcription factor ATF plays a vital role in this process.	^43^
2017	Roussel	He used CyTOF to identify markers of mononuclear phagocytic system activation in response to various stimuli and found cells that could express both M1 and M2 markers.	^44^
2019	Zilionis	He discovered the presence of macrophages in the lungs of mice that express both the M1 and M2 markers.	^45^

Abbreviations: CyTOF, mass cytometry by time of flight; HIF, hypoxia‐inducible factor.

#### 2.1.1 M1 macrophages

M1 macrophages, also referred to as classically activated or proinflammatory macrophages, are induced by exposure to bacterial products like LPS and inflammatory cytokines such as IFN‐γ and TNF‐α.^46^, ^47^ This activation state is characterized by the expression of specific surface markers, including CD80, CD86, and major histocompatibility complex (MHC) class II molecules, and the production of proinflammatory cytokines and mediators.^48^, ^49^ Key markers and functions of M1 macrophages include (a) *Cytokine production*: M1 macrophages secrete high levels of proinflammatory cytokines such as TNF‐α, IL‐1 beta (IL‐1β), IL‐6, IL‐12, and IL‐23. These cytokines orchestrate the inflammatory response, recruit and activate other immune cells, and promote tissue damage.^50^, ^51^, ^52^, ^53^ (b) *Microbicidal activity*: M1 macrophages are equipped with potent microbicidal mechanisms, including the production of reactive oxygen species (ROS), reactive nitrogen species (RNS), and the expression of inducible nitric oxide synthase (iNOS).^54^, ^55^ These factors contribute to the killing of invading pathogens and tumor cells. (c) *Antigen presentation*: M1 macrophages upregulate the expression of^56^ class II molecules, enabling them to effectively present antigens to T cells and initiate adaptive immune responses.^57^, ^58^ (d) *Tissue remodeling*: M1 macrophages secrete proteolytic enzymes, such as matrix metalloproteinases (MMPs), which contribute to the breakdown of extracellular matrix components, facilitating tissue remodeling and repair.^59^, ^60^, ^61^ M1 macrophages play crucial roles in the initial stages of inflammation, pathogen clearance, and tissue injury, coordinating the innate immune response and initiating the repair process.^62^, ^63^, ^64^

#### 2.1.2 M2 macrophages

In contrast to the proinflammatory M1 phenotype, M2 macrophages, also known as alternatively activated or anti‐inflammatory macrophages, are induced by exposure to cytokines such as IL‐4 and IL‐13 and immunomodulatory molecules like glucocorticoids and IL‐10.^65^, ^66^, ^67^, ^68^, ^69^, ^70^, ^71^, ^72^, ^73^ M2 macrophages are characterized by the expression of specific surface markers, including CD163 and CD206 (mannose receptor), and the production of anti‐inflammatory cytokines and mediators involved in tissue repair and homeostasis.^74^, ^75^, ^76^, ^77^ Key markers and functions of M2 macrophages include (a) *anti‐inflammatory cytokine production*: M2 macrophages secrete high levels of anti‐inflammatory cytokines such as IL‐10 and TGF‐β, which help to dampen the inflammatory response and promote tissue repair.^78^, ^79^, ^80^, ^81^, ^82^, ^83^ (b) *Extracellular matrix remodeling*: M2 macrophages contribute to synthesizing and remodeling extracellular matrix components, including collagen, fibronectin, and proteoglycans, facilitating tissue repair and wound healing.^84^, ^85^, ^86^ (c) *Angiogenesis*: M2 macrophages secrete proangiogenic factors, such as VEGF, which promote the formation of new blood vessels, ensuring adequate nutrient and oxygen supply to the injured tissue.^87^, ^88^, ^89^ (d) *Phagocytosis and efferocytosis*: M2 macrophages efficiently phagocytose cellular debris and apoptotic cells, a process known as efferocytosis, which is crucial for the resolution of inflammation and tissue remodeling.^90^, ^91^, ^92^, ^93^ (e) *Tissue homeostasis*: M2 macrophages play essential roles in maintaining tissue homeostasis by regulating metabolic processes, promoting insulin sensitivity, and contributing to the clearance of cellular debris.^94^, ^95^, ^96^ M2 macrophages are instrumental in the later stages of tissue repair, promoting the resolution of inflammation, angiogenesis, extracellular matrix remodeling, and restoring tissue homeostasis.^97^, ^98^, ^99^

It is important to note that the M1/M2 polarization represents a continuum, and macrophages can exhibit a range of phenotypes between these two extremes, depending on the specific microenvironmental cues they encounter.^100^, ^101^, ^102^ Additionally, recent research has revealed the existence of distinct subpopulations within the M1 and M2 categories, each with unique transcriptional profiles and functional characteristics.^68^, ^103^, ^104^, ^105^ The dynamic interplay between M1 and M2 macrophages is crucial for orchestrating an effective immune response, balancing inflammation and tissue repair, and maintaining homeostasis. Dysregulation of this balance can contribute to the development and progression of various pathological conditions, including chronic inflammatory diseases, autoimmune disorders, and impaired wound healing.^106^, ^107^, ^108^ Understanding the molecular mechanisms governing macrophage polarization and the signaling pathways that regulate their phenotypic transformation is essential for developing targeted therapeutic strategies to modulate macrophage function and enhance tissue repair and regeneration.

### 2.2 The spectrum of macrophage activation states: beyond M1/M2 dichotomy

The advent of single‐cell technologies, such as single‐cell RNA sequencing (scRNA‐seq) and mass cytometry (CyTOF), has revolutionized our understanding of macrophage heterogeneity.^109^, ^110^, ^111^ These high‐resolution techniques have allowed researchers to profile the transcriptomes and proteomes of individual macrophages, uncovering a continuum of activation states that extend beyond the M1/M2 dichotomy.^112^ A seminal study by Xue et al.^41^ used scRNA‐seq to analyze human macrophages stimulated with 28 different activation conditions. They identified 49 distinct macrophage subsets, each with a unique transcriptional signature, highlighting the incredible diversity of macrophage responses to environmental cues. Similarly, a CyTOF study by Roussel et al.^44^ demonstrated that human macrophages exhibit a spectrum of activation states in response to various stimuli, with some cells coexpressing both M1 and M2 markers. Recent studies have also revealed novel macrophage subsets with unique functions. For example, Angel et al.^113^ identified a population of antigen‐presenting macrophages in human lymph nodes that express high levels of MHC class II and costimulatory molecules, suggesting a role in adaptive immunity. Another study by Zilionis et al.^45^ discovered a subset of mouse lung macrophages expressing M1 and M2 markers that play a critical role in maintaining lung homeostasis. These findings underscore the limitations of the M1/M2 classification system and emphasize the need for a more nuanced understanding of macrophage plasticity. The spectrum of activation states revealed by single‐cell technologies highlights the remarkable adaptability of macrophages to diverse environmental cues and their multifaceted roles in health and disease.^8^, ^114^, ^115^

### 2.3 Tissue‐specific imprinting of macrophage identity and function

One of the most significant advances in macrophage biology over the past decade has been recognizing the profound influence of tissue microenvironments on macrophage development, phenotype, and function. Macrophages are present in virtually all tissues, performing specialized functions tailored to the unique demands of their local niche.^116^ Recent studies have shown that tissue‐specific factors, such as cytokines, metabolites, and cell–cell interactions, can imprint distinct transcriptional and epigenetic signatures on resident macrophages, giving rise to specialized subsets with unique functions.^117^, ^118^ For example, Lavin et al.^40^ demonstrated that macrophages from different tissues, such as the lung, liver, and spleen, possess distinct enhancer landscapes shaped by tissue‐specific transcription factors. The gut microbiome has also emerged as a critical regulator of intestinal macrophage function.^119^, ^120^ Studies have shown that microbial metabolites, such as short‐chain fatty acids and taurine, can modulate the phenotype and activity of intestinal macrophages, promoting homeostasis and protecting against enteric infections.^121^, ^122^, ^123^ These findings highlight the importance of studying macrophages in their native tissue context and underscore the limitations of extrapolating conclusions from in vitro studies to in vivo settings. The tissue‐specific imprinting of macrophage identity and function has important implications for our understanding of immune regulation and disease pathogenesis, as dysregulation of these processes may contribute to developing inflammatory and metabolic disorders.^116^, ^124^, ^125^

### 2.4 Ontogenetic diversity of tissue‐resident macrophages

Another significant paradigm shift in macrophage biology has been the discovery of the ontogenetic diversity of tissue‐resident macrophages (TrMΦ).^126^, ^127^ Contrary to the traditional view that all tissue macrophages are derived from circulating monocytes, recent fate mapping studies have revealed that many TrMΦ are established during embryonic development and maintain themselves through local proliferation, independent of adult monocyte input.^128^, ^129^ For example, microglia, the resident macrophages of the central nervous system, have been shown to originate from yolk sac‐derived progenitors that seed the brain early in embryonic development.^130^ Similarly, Kupffer cells, the resident macrophages of the liver, are derived from a combination of yolk sac and fetal liver progenitors.^131^ The ontogenetic origin of TrMΦ has important implications for their function and response to environmental challenges. Embryonically derived macrophages have been shown to possess unique transcriptional and epigenetic profiles compared with their monocyte‐derived counterparts, which may confer distinct functional properties.^132^, ^133^ The discovery of the ontogenetic diversity of TrMΦ has also prompted a reevaluation of the contribution of monocyte‐derived macrophages to tissue homeostasis and inflammation. While monocyte‐derived macrophages play a crucial role in the response to injury and infection, their contribution to the maintenance of TrMΦ populations appears context dependent. It may vary across different organs and disease states.^134^, ^135^

### 2.5 Trained immunity: long‐term reprogramming of macrophages

In addition to short‐term plasticity, macrophages can undergo long‐term functional reprogramming in response to microbial stimuli, known as trained innate immunity.^136^, ^137^ This process involves epigenetic and metabolic changes that enhance the responsiveness of macrophages to subsequent challenges, providing a form of innate immune memory.^138^, ^139^ A landmark study by Quintin et al.^39^ demonstrated that exposure to the fungal cell wall component β‐glucan induces epigenetic modifications in human monocytes, leading to increased production of proinflammatory cytokines upon restimulation. This trained immunity is mediated by changes in histone methylation, acetylation, and a metabolic shift toward glycolysis.^140^, ^141^ Subsequent studies have shown that other microbial stimuli, such as the bacillus Calmette‐Guérin (BCG) vaccine and the bacterial component muramyl dipeptide, can also induce trained immunity in macrophages.^139^, ^142^, ^143^, ^144^ Arts et al.^43^ identified a critical role for ATF7 in mediating the epigenetic reprogramming of macrophages during β‐glucan‐induced training. Another study by Cheng et al.^42^ demonstrated that the metabolic enzyme glutamine synthetase is essential for the induction of trained immunity by β‐glucan, highlighting the link between metabolism and epigenetic reprogramming. The discovery of trained immunity has important implications for developing novel immunotherapies. For example, Moorlag et al.^145^ showed that BCG vaccination induces trained immunity in human monocytes, enhancing their ability to eliminate the respiratory syncytial virus. Harnessing trained immunity could be a promising strategy for boosting host defense against infectious diseases.

In summary, the study of macrophage plasticity has come a long way since the initial discovery of these versatile immune cells by Élie Metchnikoff in 1882. The traditional M1/M2 classification system provided a valuable framework for understanding macrophage polarization, but recent advances have revealed a spectrum of activation states that extend beyond this dichotomy. Single‐cell technologies have uncovered macrophages’ remarkable heterogeneity and ability to adapt to diverse tissue microenvironments. The functional significance of macrophage plasticity is evident in their roles in maintaining tissue homeostasis, orchestrating immune responses, and contributing to disease pathogenesis. Tissue‐specific imprinting and trained immunity further highlight the adaptability of macrophages to their local environment and their capacity for long‐term functional reprogramming.

## 3 SIGNALING PATHWAYS ORCHESTRATING MACROPHAGE POLARIZATION: AN INTRICATE REGULATORY NETWORK

Macrophages are versatile innate immune cells that play critical roles in host defense, tissue homeostasis, and disease pathogenesis. These cells exhibit remarkable plasticity, adapting their phenotype and function in response to diverse microenvironmental signals. Macrophage polarization refers to the process by which macrophages acquire distinct functional programs, classically categorized into two main subsets: classically activated (M1) and alternatively activated (M2) macrophages. M1 macrophages are induced by Th1 cytokines, such as IFN‐γ, and microbial products, including LPS. They exhibit potent proinflammatory and microbicidal activities, secreting high levels of proinflammatory cytokines (e.g., IL‐1β, IL‐6, IL‐12, and TNF‐α) and producing reactive oxygen and nitrogen species. In contrast, M2 macrophages are polarized by Th2 cytokines, such as IL‐4 and IL‐13, associated with anti‐inflammatory responses, tissue repair, and tumor progression. M2 macrophages produce anti‐inflammatory cytokines (e.g., IL‐10 and TGF‐β) and express scavenger receptors, mannose receptors, and ARG‐1. Recent studies have revealed that macrophage polarization is a highly dynamic and complex process involving the integration of multiple signaling pathways. These pathways are triggered by the engagement of PRRs, cytokine receptors, and other surface molecules, activating transcription factors and epigenetic regulators that shape the macrophage transcriptional landscape. Understanding the molecular mechanisms governing macrophage polarization is crucial for developing targeted therapies to modulate macrophage function in various pathological conditions, such as inflammatory diseases and cancer. This section provides an in‐depth discussion of the critical signaling pathways that orchestrate macrophage polarization, focusing on recent findings and their implications for therapeutic interventions. We will explore the roles of toll‐like receptors (TLRs), STAT proteins, nuclear receptors, miRNAs, metabolic reprogramming, and epigenetic modifications in shaping macrophage activation states. Furthermore, we will highlight the crosstalk between these signaling cascades and their potential as therapeutic targets for modulating macrophage function in disease contexts.

### 3.1 Toll‐like receptors: sentinels of macrophage polarization

TLRs are a family of pattern recognition receptors (PRRs) that play a pivotal role in the innate immune response by recognizing conserved molecular patterns associated with pathogens (PAMPs) and endogenous danger signals (DAMPs).^146^, ^147^ TLR signaling is a crucial driver of macrophage polarization, particularly in M1 activation.^148^, ^149^, ^150^ Engagement of TLRs by their respective ligands triggers the recruitment of adaptor proteins, such as myeloid differentiation primary response 88 (MyD88) and TIR‐domain‐containing adapter‐inducing IFN‐β (TRIF), which initiate downstream signaling cascades.^151^, ^152^, ^153^, ^154^ These cascades lead to the activation of transcription factors, including nuclear factor‐κB (NF‐κB), activator protein‐1 (AP‐1), and IFN regulatory factors (IRFs), which drive the expression of proinflammatory genes and shape the M1 macrophage phenotype.^155^, ^156^, ^157^, ^158^, ^159^, ^160^ TLR4, the receptor for bacterial LPS, is a potent inducer of M1 polarization.^159^, ^161^ Upon LPS recognition, TLR4 activates both MyD88‐dependent and TRIF‐dependent pathways, producing proinflammatory cytokines and type I IFNs, respectively.^162^, ^163^ The MyD88‐dependent pathway involves the activation of NF‐κB and mitogen‐activated protein kinases (MAPKs), such as p38, JNK, and ERK, which promote the expression of proinflammatory genes.^164^, ^165^, ^166^ The TRIF‐dependent pathway, on the other hand, activates IRF3 and IRF7, leading to the production of type I IFNs and the induction of IFN‐stimulated genes.^167^, ^168^, ^169^ Other TLRs, such as TLR2 (which recognizes bacterial lipoproteins) and TLR3 (which detects viral double‐stranded RNA), also contribute to macrophage polarization. TLR2 signaling predominantly activates NF‐κB and MAPKs, driving M1 polarization, while TLR3 activation leads to the production of type I IFNs and proinflammatory cytokines via the TRIF‐dependent pathway.^170^, ^171^, ^172^ Recent studies have revealed that TLR signaling can also modulate M2 polarization. For instance, activation of TLR2 and TLR4 has been shown to enhance the expression of M2 markers, such as ARG‐1 and Ym1, in the presence of IL‐4.^150^, ^173^ This suggests that TLR signaling can fine‐tune macrophage polarization depending on the microenvironmental context and the presence of other polarizing stimuli. Targeting TLR signaling pathways has emerged as a promising therapeutic strategy for modulating macrophage polarization in various disease settings. For example, inhibition of TLR4 signaling has been shown to attenuate M1 polarization and promote M2‐like phenotypes in models of inflammatory diseases, such as rheumatoid arthritis and inflammatory bowel disease.^174^, ^175^, ^176^ Conversely, activation of TLR3 signaling has been explored to boost antitumor immunity by promoting M1 polarization in tumor‐associated macrophages (TAMs).^177^, ^178^

### 3.2 STAT signaling: a key regulator of macrophage polarization

STAT proteins are transcription factors that play critical roles in cytokine signaling and macrophage polarization.^179^ Different STAT proteins are activated by specific cytokines and regulate distinct aspects of macrophage function, with STAT1 and STAT6 being particularly important for M1 and M2 polarization, respectively.^180^ STAT1 is activated by IFN‐γ, a potent inducer of M1 polarization.^181^, ^182^ Upon IFN‐γ binding to its receptor, Janus kinases (JAKs) are activated, leading to the phosphorylation and dimerization of STAT1. Activated STAT1 dimers translocate to the nucleus, where they bind to gamma‐activated sequences in the promoters of target genes, driving the expression of proinflammatory and microbicidal factors, such as inducible iNOS and IL‐12.^182^, ^183^ In contrast, STAT6 is activated by the Th2 cytokines IL‐4 and IL‐13, critical drivers of M2 polarization.^184^, ^185^ Engagement of IL‐4 or IL‐13 with their respective receptors leads to the activation of JAKs and the phosphorylation of STAT6. Phosphorylated STAT6 dimers translocate to the nucleus and bind to specific DNA sequences, promoting the expression of M2‐associated genes, such as ARG‐1, mannose receptor (CD206), and resistin‐like molecule‐α (FIZZ1).^185^, ^186^ The balance between STAT1 and STAT6 activation is a critical determinant of macrophage polarization, with the relative abundance of IFN‐γ and IL‐4/IL‐13 in the microenvironment playing a key role.^187^, ^188^ Interestingly, STAT1 and STAT6 have been shown to antagonize each other's functions, with STAT1 activation suppressing M2 polarization and STAT6 activation inhibiting M1 responses.^189^, ^190^ This antagonism highlights the complex interplay between signaling pathways in shaping macrophage activation states. Targeting STAT signaling pathways has emerged as a potential therapeutic strategy for modulating macrophage polarization in various disease contexts. For example, inhibition of STAT1 signaling has been explored to attenuate M1 polarization and promote tissue repair in models of inflammatory diseases, such as multiple sclerosis and inflammatory bowel disease.^189^, ^191^ Conversely, activation of STAT6 signaling has been investigated as a potential approach to promote M2 polarization and resolve inflammation in conditions such as obesity and atherosclerosis.^192^, ^193^

### 3.3 Nuclear receptors: transcriptional regulators of macrophage polarization

Nuclear receptors are a family of ligand‐activated transcription factors that regulate macrophage polarization and function.^194^ Two nuclear receptors, peroxisome proliferator‐activated receptor‐γ (PPARγ) and liver X receptors (LXRs), have been particularly implicated in modulating macrophage activation states. PPARγ is a master regulator of M2 polarization, promoting the expression of anti‐inflammatory and tissue repair genes.^195^ Activation of PPARγ by endogenous ligands, such as polyunsaturated fatty acids and eicosanoids, or synthetic agonists, such as thiazolidinediones, leads to the formation of heterodimers with retinoid X receptors.^196^ These heterodimers bind to specific DNA sequences called PPAR response elements in the promoters of target genes, driving the expression of M2‐associated factors, such as ARG‐1, CD206, and IL‐10.^197^ PPARγ activation has been shown to antagonize M1 polarization by inhibiting the activity of proinflammatory transcription factors, such as NF‐κB and AP‐1.^198^ This antagonism is mediated through various mechanisms, including direct protein–protein interactions, competition for coactivators, and induction of anti‐inflammatory genes. Consequently, PPARγ agonists have been explored as potential therapeutic agents for modulating macrophage polarization in inflammatory diseases, such as atherosclerosis, obesity, and insulin resistance.^198^, ^199^, ^200^ LXRs, including LXRα and LXRβ, are another nuclear receptor class regulating macrophage polarization and function. LXRs are activated by oxysterols and oxidized cholesterol derivatives and play critical roles in lipid metabolism and inflammation. Activation of LXRs has been shown to promote an anti‐inflammatory M2‐like phenotype in macrophages, characterized by increased expression of genes involved in lipid efflux, such as ATP‐binding cassette transporters A1 and G1, and reduced production of proinflammatory cytokines.^201^, ^202^ LXR agonists have demonstrated anti‐inflammatory and immunomodulatory effects in various disease models, including atherosclerosis, Alzheimer's, and autoimmune disorders.^203^, ^204^ These effects are mediated, in part, by the ability of LXRs to inhibit NF‐κB signaling and promote the resolution of inflammation.^205^ As such, targeting LXR signaling has emerged as a potential therapeutic strategy for modulating macrophage polarization and function in inflammatory diseases.^206^

### 3.4 MicroRNAs: posttranscriptional regulators of macrophage polarization

miRNAs are small noncoding RNAs that regulate gene expression at the posttranscriptional level by binding to complementary sequences in the 3′ untranslated regions of target mRNAs, leading to their degradation or translational repression.^207^, ^208^, ^209^ Evidence suggests that miRNAs play crucial roles in regulating macrophage polarization and function. Several miRNAs have been identified as key regulators of M1 polarization, including miR‐155, miR‐125b, and miR‐146a.^210^, ^211^, ^212^ miR‐155 is upregulated in M1 macrophages and promotes the expression of proinflammatory genes by targeting negative regulators of NF‐κB signaling, such as suppressor of cytokine signaling 1 (SOCS1) and Src homology 2 domain‐containing inositol‐5‐phosphatase 1.^213^, ^214^ miR‐125b, on the other hand, inhibits M1 polarization by targeting the transcription factor IRF4, which is involved in the induction of proinflammatory cytokines.^215^ miR‐146a acts as a negative feedback regulator of M1 responses by targeting key components of the NF‐κB signaling pathway, such as IL‐1 receptor‐associated kinase 1 and TNF receptor‐associated factor 6.^216^ Similarly, several miRNAs have been implicated in regulating M2 polarization, including miR‐21, miR‐124, and miR‐223.^217^ miR‐21 promotes M2 polarization by targeting programmed cell death 4 (PDCD4), a negative regulator of IL‐10 production. miR‐124 is upregulated in M2 macrophages and promotes the expression of M2‐associated genes, such as ARG‐1 and FIZZ1, by targeting the transcription factor CCAAT/enhancer‐binding protein‐α.^218^ miR‐223 has been shown to promote M2 polarization by targeting Pknox1, a transcription factor that suppresses the expression of M2‐associated genes.^219^, ^220^ The therapeutic potential of targeting miRNAs to modulate macrophage polarization has been explored in various disease models. For example, inhibition of miR‐155 has been shown to attenuate M1 polarization and promote M2‐like phenotypes in models of inflammatory diseases, such as rheumatoid arthritis and.^221^, ^222^ Conversely, overexpression of miR‐21 or miR‐124 has been investigated to promote M2 polarization and resolve inflammation in conditions such as sepsis and spinal cord injury.^223^

### 3.5 Metabolic regulation of macrophage polarization

Macrophage polarization is closely linked to metabolic reprogramming, with distinct metabolic profiles associated with M1 and M2 phenotypes.^5^, ^224^, ^225^ M1 macrophages rely on glycolysis and the pentose phosphate pathway to meet their energy demands and support their proinflammatory functions.^226^ In contrast, M2 macrophages primarily utilize oxidative phosphorylation and fatty acid oxidation for energy production.^227^ The mechanistic target of the rapamycin (mTOR) pathway is a central regulator of macrophage metabolism and polarization. mTOR complex 1 (mTORC1) is activated in M1 macrophages and promotes glycolysis through the induction of hypoxia‐inducible factor‐1α (HIF‐1α) and the expression of glycolytic enzymes.^228^, ^229^, ^230^, ^231^ Inhibition of mTORC1 by rapamycin or genetic deletion of its component Raptor skews macrophages toward an M2 phenotype.^232^ Adenosine monophosphate‐activated protein kinase (AMPK), a key energy sensor, is crucial in regulating macrophage polarization.^233^ AMPK activation promotes M2 polarization by inhibiting mTORC1 and enhancing oxidative metabolism.^234^, ^235^ Metformin, an AMPK activator, has been shown to promote M2 polarization and alleviate inflammatory responses in various disease models.^236^, ^237^ Recent studies have also highlighted the role of lipid metabolism in macrophage polarization. Fatty acid synthesis is upregulated in M1 macrophages, while fatty acid oxidation is associated with M2 polarization.^238^, ^239^ PPARs, particularly PPARγ and PPARδ, are key regulators of lipid metabolism and have been implicated in promoting M2 polarization.^240^

### 3.6 Epigenetic regulation of macrophage polarization

Epigenetic modifications, such as DNA methylation and histone modifications, are crucial in regulating macrophage polarization by modulating the accessibility of polarization‐associated genes.^4^, ^240^ M1 and M2 macrophages exhibit distinct epigenetic signatures contributing to their phenotypic stability and plasticity. Histone deacetylases (HDACs) have emerged as essential regulators of macrophage polarization.^241^, ^242^ HDAC3 has been shown to promote M1 polarization by deacetylating and activating NF‐κB, while its inhibition skews macrophages toward an M2 phenotype.^243^, ^244^, ^245^ In contrast, HDAC4 and HDAC5 have been implicated in promoting M2 polarization through the deacetylation of STAT6.^246^, ^247^, ^248^, ^249^ DNA methylation also plays a role in macrophage polarization. The DNA methyltransferase DNMT3b is upregulated in M1 macrophages and mediates the silencing of M2‐associated genes.^250^, ^251^ Conversely, the demethylase TET2 promotes M2 polarization by demethylating and activating M2‐associated genes.^252^ Noncoding RNAs, such as miRNAs and long noncoding RNAs (lncRNAs), have also emerged as critical epigenetic regulators of macrophage polarization.^253^ For example, miR‐21 promotes M1 polarization by targeting the anti‐inflammatory cytokine IL‐10, while miR‐146a promotes M2 polarization by inhibiting NF‐κB signaling.^216^, ^254^ LncRNAs, such as lncRNA‐Cox2 and lncRNA‐Mirt2, have been shown to regulate macrophage polarization by modulating the expression of polarization‐associated genes^255^, ^256^ (Table 2).

**TABLE 2:** Factors that regulate macrophage polarization.

Macrophage polarization	Extracellular stimulation		transcription factor	Epigenetic regulation					Metabolic regulation
M1 polarization	Stimulus	Receptors	NF‐κB IRF1^257^ IRF5 IRF8 STAT1^258^ AP‐1^259^ ZHX2^260^	DNA methylation	Histone acetylation	Histone deacetylation	Histone demethylation	Noncoding RNA	Anaerobic glycolysis iNOS synthesis Iron supplement^261^
	LPS	TLR4		DNMT1^262^, ^263^ DNMT3^250^	PCAF^264^	HDAC3^265^ HDAC8^266^ HDAC5^248^ HDAC7^267^ HDAC9^268^	JMJD1C^269^	miR‐9‐5p^270^ miR ‐127^271^ miR125b^215^ miR‐155^272^ miR‐146^53^ miR‐let‐7a/f miR‐378M1 miR‐302a	
	IFN‐γ	IFN‐γR							
	IFN‐β	IFN‐αR IFN‐βR							
	TNF‐α	TNFR1/2							
	PAMPs DAMPs	NLR							
	GM‐CSF	CSF2Rα							
M2 polarization	IL‐4 IL‐13	IL‐4Rα	STAT6^273^ STAT3^274^ c‐Maf^275^ IRF‐3 IRF‐4	–	H3Ac^276^ MOF^277^	HDAC1^278^ HDAC2^279^ HDAC6^280^ HDAC10^281^ HDAC4^282^	UTX^283^ KDM3c^284^	miR‐let‐7c/e^285^ miR‐21‐5p^286^ miR‐27a^287^ miR‐181^288^ miR‐147^289^ miR‐124‐3p^217^ miR‐132^290^ miR‐146a^216^	Glucose aerobic oxidation^291^ Fatty acid oxidation^292^ ARG‐1 synthesis^293^ Iron deprivation^294^
	TGF‐β	TβRI TβRII							
	Glucocorticoid	IFN‐αR IFN‐βR							
	IL‐10	IL‐10R							

Abbreviations: DAMP, pathogen‐associated molecular patterns; DNMT, DNA methyltransferase; GM‐CSF, granulocyte‐macrophage colony‐stimulating factor; HDAC, histone deacetylase; INF, interferon; LPS, lipopolysaccharide; miRNA, microRNA; ARG‐1, arginase‐1; PAMPs, pathogen‐associated molecular patterns; PCAF, P300/CBP‐associated factor; TNF, tumor necrosis factor; TGF, transforming growth factor; UTX, ubiquitously transcribed tetratricopeptide repeat on chromosome X.

### 3.7 Polarization of macrophages in some pathological processes

Macrophage polarization is crucial in various physiological and pathological processes, including host defense, tissue homeostasis, inflammatory diseases, and cancer. Understanding the signaling pathways that govern macrophage polarization can provide valuable insights into disease pathogenesis and guide the development of targeted therapies. In inflammatory diseases, such as rheumatoid arthritis and inflammatory bowel disease, an imbalance between M1 and M2 macrophages contributes to chronic inflammation and tissue damage.^295^, ^296^, ^297^, ^298^ Targeting the signaling pathways that promote M1 polarization, such as TLR and NF‐κB signaling, has shown promise in alleviating inflammatory responses in preclinical models. Conversely, promoting M2 polarization through activating STAT6 or PPARγ has been explored as a strategy to resolve inflammation and promote tissue repair. In cancer, TAMs often exhibit an M2‐like phenotype and contribute to tumor progression by promoting angiogenesis, immunosuppression, and metastasis.^299^ Targeting the signaling pathways that drive M2 polarization in TAMs, such as colony‐stimulating factor (CSF)‐1/CSF‐1R and IL‐4/IL‐13 signaling, has emerged as a promising therapeutic approach.^300^, ^301^ Reprogramming TAMs toward an M1‐like phenotype through TLR or STING signaling activation has also shown potential in enhancing antitumor immunity.^302^, ^303^ In tissue regeneration and wound healing, M2 macrophages are crucial in promoting tissue repair and resolving inflammation. Harnessing the signaling pathways that promote M2 polarization, such as IL‐4/STAT6 and IL‐10/STAT3 signaling, has been explored to enhance tissue regeneration and limit fibrosis.^304^, ^305^, ^306^

In summary, macrophage polarization is a dynamic and finely tuned process orchestrated by a complex network of signaling pathways. The integration of signals from TLRs, cytokines, and metabolic pathways shapes the functional phenotype of macrophages, allowing them to adapt to various microenvironmental cues. Recent advances in understanding the molecular mechanisms governing macrophage polarization have provided valuable insights into the role of these cells in health and disease. However, several challenges and opportunities remain in the field of macrophage polarization. The dichotomous M1/M2 classification, while applicable as a conceptual framework, oversimplifies the spectrum of macrophage activation states. Future studies should focus on delineating the complex heterogeneity of macrophage phenotypes and their functional implications in specific tissue contexts. Moreover, the crosstalk between signaling pathways and the influence of the tissue microenvironment on macrophage polarization warrants further investigation. Integrating multiomics approaches, such as transcriptomics, proteomics, and metabolomics, can provide a comprehensive understanding of the regulatory networks governing macrophage polarization. Translating the knowledge of macrophage polarization signaling into clinical applications remains a major challenge. Developing targeted therapies that modulate specific signaling pathways in macrophages while minimizing off‐target effects is crucial. Nanoparticle‐based drug delivery systems and engineered exosomes have shown promise in selectively targeting macrophages and modulating their polarization state.

## 4 MACROPHAGES: ORCHESTRATORS OF TISSUE REPAIR AND REGENERATION

Tissue injury triggers a highly coordinated cascade of events aimed at restoring tissue integrity and function. At the forefront of this intricate process are macrophages, versatile immune cells that exhibit remarkable plasticity and functional diversity. These cells play pivotal roles throughout the distinct phases of tissue repair and regeneration, seamlessly transitioning between proinflammatory and anti‐inflammatory phenotypes to facilitate the progression from initial injury to complete tissue restoration.

### 4.1 The inflammatory phase: M1 macrophages as first responders

#### 4.1.1 Initiation of the inflammatory response

The inflammatory phase is initiated by recognizing DAMPs and PAMPs by PRRs on macrophages and other immune cells.^307^, ^308^ This recognition triggers a rapid phenotypic transformation of macrophages from a resting state to an activated, proinflammatory state, known as the M1 phenotype. The activation of M1 macrophages is mediated by a diverse array of PRRs, including TLRs, NLRs, and RLRs.^309^, ^310^ TLRs, such as TLR4 and TLR2, are particularly crucial in this process, initiating signaling cascades that converge on the activation of transcription factors like NF‐κB and AP‐1, driving the expression of proinflammatory genes.^309^, ^311^ Upon activation, M1 macrophages rapidly produce and secrete various proinflammatory cytokines and chemokines, including IL‐1β, IL‐6, IL‐12, IL‐23, and TNF‐α.^22^, ^312^, ^313^ These mediators orchestrate the inflammatory response, recruiting additional immune cells to the injury site and amplifying the inflammatory cascade.

#### 4.1.2 Pathogen clearance and antimicrobial effector mechanisms

During the inflammatory phase, a primary function of M1 macrophages is the clearance of pathogens through various antimicrobial effector mechanisms. Phagocytosis, the process by which macrophages engulf and internalize pathogens or cellular debris, is a critical component of the innate immune response.^314^, ^315^, ^316^ M1 macrophages express receptors such as Fc and complement receptors, facilitating the recognition and binding of opsonized pathogens for efficient phagocytosis.^317^, ^318^ Once internalized, pathogens are subjected to a range of intracellular killing mechanisms within the phagolysosome, a specialized compartment formed by the fusion of the phagosome with lysosomes. These mechanisms include: (a) *ROS production*: M1 macrophages generate a potent oxidative burst through the activity of NADPH oxidase, producing superoxide radicals and other ROS that can directly damage and kill pathogens.^309^, ^311^ (b) *NO production*: iNOS in M1 macrophages catalyzes the production of NO, a highly reactive free radical that can directly kill or inhibit the growth of pathogens.^319^, ^320^, ^321^ (c) *Antimicrobial peptides and enzymes*: M1 macrophages produce antimicrobial peptides, such as defensins and cathelicidins, and lysosomal enzymes, like cathepsins and lysozymes, which can disrupt and degrade microbial cell walls and membranes.^322^, ^323^, ^324^ (d) *Acidification*: The phagolysosome provides an acidic environment, with a pH ranging from 4.5 to 5, which can directly inhibit the growth and survival of many pathogens.^325^, ^326^ In addition to intracellular killing mechanisms, M1 macrophages employ extracellular strategies to combat pathogens, such as neutrophil extracellular trap formation, antimicrobial peptide and enzyme secretion, and cytokine and chemokine production to recruit and activate additional immune cells.^327^, ^328^, ^329^, ^330^

#### 4.1.3 Debris removal and tissue remodeling

**FIGURE 1:**
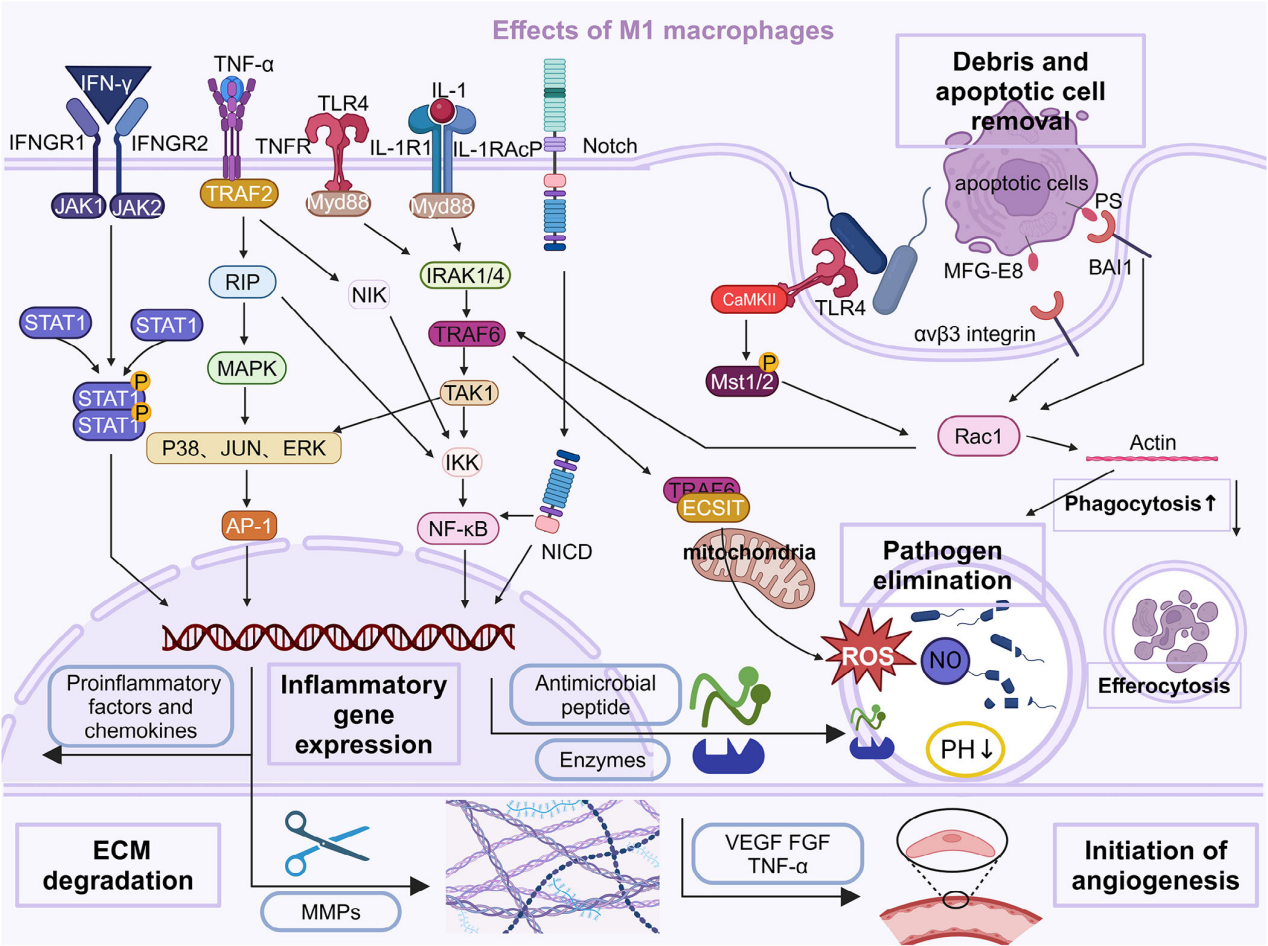
The effects of M1 macrophages in wound healing. While early in the repair process, their inflammatory actions are essential. Different PRRs form a complex network after being activated by corresponding signals, precisely coordinate the secretion of inflammatory factors, and induce the phenotype of M1 macrophages, such as stimulating microbicidal, inflammatory activation, phagocytosis, cellular burial, and other biological effects. In addition, M1 macrophages can regulate ECM degradation and initiate angiogenesis. Prolonged M1 response can lead to chronic inflammation and impaired healing. Created with BioRender.com.

M1 macrophages are crucial in removing cellular debris and initiating tissue remodeling during the inflammatory phase.^331^ They are essential for the clearance of apoptotic cells, a process known as efferocytosis, which prevents the release of potentially harmful intracellular contents and promotes the resolution of inflammation.^314^, ^332^ Furthermore, M1 macrophages initiate the remodeling of the extracellular matrix (ECM) by producing proteolytic enzymes, such as MMPs, and cytokines that regulate ECM turnover.^333^ This ECM degradation facilitates the removal of damaged or necrotic tissue and creates space for the subsequent influx of new cells and the deposition of a provisional ECM.^334^, ^335^ M1 macrophages also contribute to the initiation of angiogenesis, the formation of new blood vessels, by producing proangiogenic factors like VEGF, bFGF, and TNF‐α.^336^, ^337^ These factors stimulate endothelial cell proliferation, migration, and the assembly of functional vascular structures (Figure 1).

### 4.2 The proliferation and remodeling phase: M2 macrophages facilitate tissue regeneration

#### 4.2.1 Anti‐inflammatory signaling and resolution of inflammation

Following the initial inflammatory phase, the tissue repair process transitions into the proliferative and remodeling phases, characterized by the resolution of inflammation, angiogenesis, and ECM deposition and remodeling.^62^, ^68^ During this stage, M2 macrophages, also known as alternatively activated or anti‐inflammatory macrophages, play a crucial role in orchestrating these processes and facilitating tissue regeneration. One of the critical functions of M2 macrophages is to promote the resolution of inflammation and create an environment conducive to tissue repair. They achieve this through the production of various anti‐inflammatory mediators and the suppression of proinflammatory pathways. M2 macrophages secrete anti‐inflammatory cytokines that counteract the proinflammatory effects of M1 macrophages and other immune cells. The primary anti‐inflammatory cytokine produced by M2 macrophages is IL‐10, which has potent immunosuppressive properties.^338^, ^339^ IL‐10 inhibits the production of proinflammatory cytokines, such as TNF‐α, IL‐1β, and IL‐6, by M1 macrophages and other immune cells and downregulates the expression of MHC class II molecules and costimulatory molecules on antigen‐presenting cells, thereby suppressing the activation and proliferation of T cells.^340^, ^341^, ^342^ In addition to IL‐10, M2 macrophages produce TGF‐β, which has anti‐inflammatory and immunosuppressive effects.^343^ TGF‐β inhibits the activation and proliferation of T cells, suppresses the production of proinflammatory cytokines, and promotes the differentiation of regulatory T cells, which play a crucial role in maintaining immune homeostasis and resolving inflammation.^344^ M2 macrophages employ various mechanisms to suppress proinflammatory signaling pathways and attenuate the inflammatory response. One key mechanism is the upregulation of negative regulators of inflammation, such as SOCS proteins and A20 (TNFAIP3). SOCS proteins inhibit the JAK–STAT signaling pathway, which produces proinflammatory cytokines, while A20 negatively regulates the NF‐κB signaling pathway, a central regulator of inflammation.^345^, ^346^, ^347^ Additionally, M2 macrophages produce anti‐inflammatory lipid mediators, such as lipoxins, resolvins, and protectins, which can actively suppress proinflammatory signaling pathways and promote the clearance of apoptotic cells and debris.^348^

#### 4.2.2 Angiogenesis and vascular remodeling

M2 macrophages promote angiogenesis and vascular remodeling during tissue repair.^349^, ^350^ They secrete various proangiogenic factors that stimulate endothelial cell proliferation, migration, and differentiation, facilitating the formation of new blood vessels. These factors include: (a) *VEGF*: a potent proangiogenic factor that induces endothelial cell proliferation, migration, and tube formation.^87^, ^351^, ^352^ (b) *bFGF*: stimulates endothelial cell proliferation and migration, as well as the production of proteolytic enzymes that facilitate cell invasion and ECM remodeling, essential processes for angiogenesis.^353^, ^354^ (c) *Placental growth factor*: a member of the VEGF family that is crucial in promoting the recruitment and differentiation of endothelial progenitor cells, contributing to the formation of new blood vessels.^355^, ^356^ (d) *Angiopoietins*: M2 macrophages produce angiopoietins, such as Ang‐1 and Ang‐2, which regulate the maturation, stabilization, and remodeling of newly formed blood vessels.^357^ In addition to producing proangiogenic factors, M2 macrophages can directly interact with endothelial cells and facilitate the formation of new blood vessels through a process known as vascular mimicry. This involves the physical association of M2 macrophages with endothelial cells, forming multicellular structures resembling vascular networks.^358^, ^359^ M2 macrophages express adhesion molecules and receptors, such as integrin αvβ3 and the Tie2 receptor, mediating their interaction with endothelial cells and enabling the exchange of proangiogenic signals and the coordination of cellular processes involved in angiogenesis. Furthermore, M2 macrophages can transdifferentiate into endothelial‐like cells, directly contributing to forming new blood vessels. This transdifferentiation is mediated by various transcription factors, such as Prox1 and Coup‐TFII, which are involved in endothelial cell differentiation and vascular development.^360^, ^361^

#### 4.2.3 Extracellular matrix remodeling and tissue regeneration

The proliferation and remodeling phase is characterized by the deposition and remodeling of the ECM, a complex network of proteins and polysaccharides that provide structural support and signaling cues for cell migration, proliferation, and differentiation.^362^, ^363^, ^364^, ^365^, ^366^ M2 macrophages play a crucial role in regulating ECM remodeling and promoting tissue regeneration through the production of ECM components, regulation of ECM‐remodeling enzymes, and modulation of fibroblast and stem cell behavior.^367^, ^368^, ^369^, ^370^

M2 macrophages contribute to the deposition and remodeling of the ECM by producing various ECM components, including: (a) *Collagens*: M2 macrophages secrete different types of collagens, such as collagen I, III, and IV, which are essential for the formation of the provisional ECM and the subsequent deposition of the mature ECM during tissue repair.^371^ (b) *Fibronectin*: a glycoprotein that plays a crucial role in cell adhesion, migration, and ECM assembly. M2 macrophages produce fibronectin, which helps create a provisional matrix for cell migration and proliferation during tissue repair.^371^ (c) *Tenascin‐C*: an ECM glycoprotein highly expressed during tissue repair that promotes cell migration, proliferation, and angiogenesis. M2 macrophages secrete tenascin‐C, which modulates the activity of various growth factors and cytokines, thereby regulating cellular processes in tissue regeneration.^372^, ^373^ (d) *GAGs*: M2 macrophages produce various GAGs, such as hyaluronic acid, heparan sulfate, and chondroitin sulfate, essential ECM components. GAGs interact with growth factors, cytokines, and ECM proteins, modulating their activity and regulating cellular processes in tissue repair and regeneration.^97^, ^374^ The deposition of a provisional ECM provides a scaffold for the recruitment and organization of various cell types, including endothelial cells, fibroblasts, and stem cells, enabling the formation of new tissue and restoring tissue integrity.^375^, ^376^, ^377^ Furthermore, the ECM components produced by M2 macrophages play a crucial role in modulating the behavior of other cells involved in tissue repair, such as cell migration, proliferation, and differentiation, through interactions with integrin receptors and the modulation of growth factor and cytokine activity.

**FIGURE 2:**
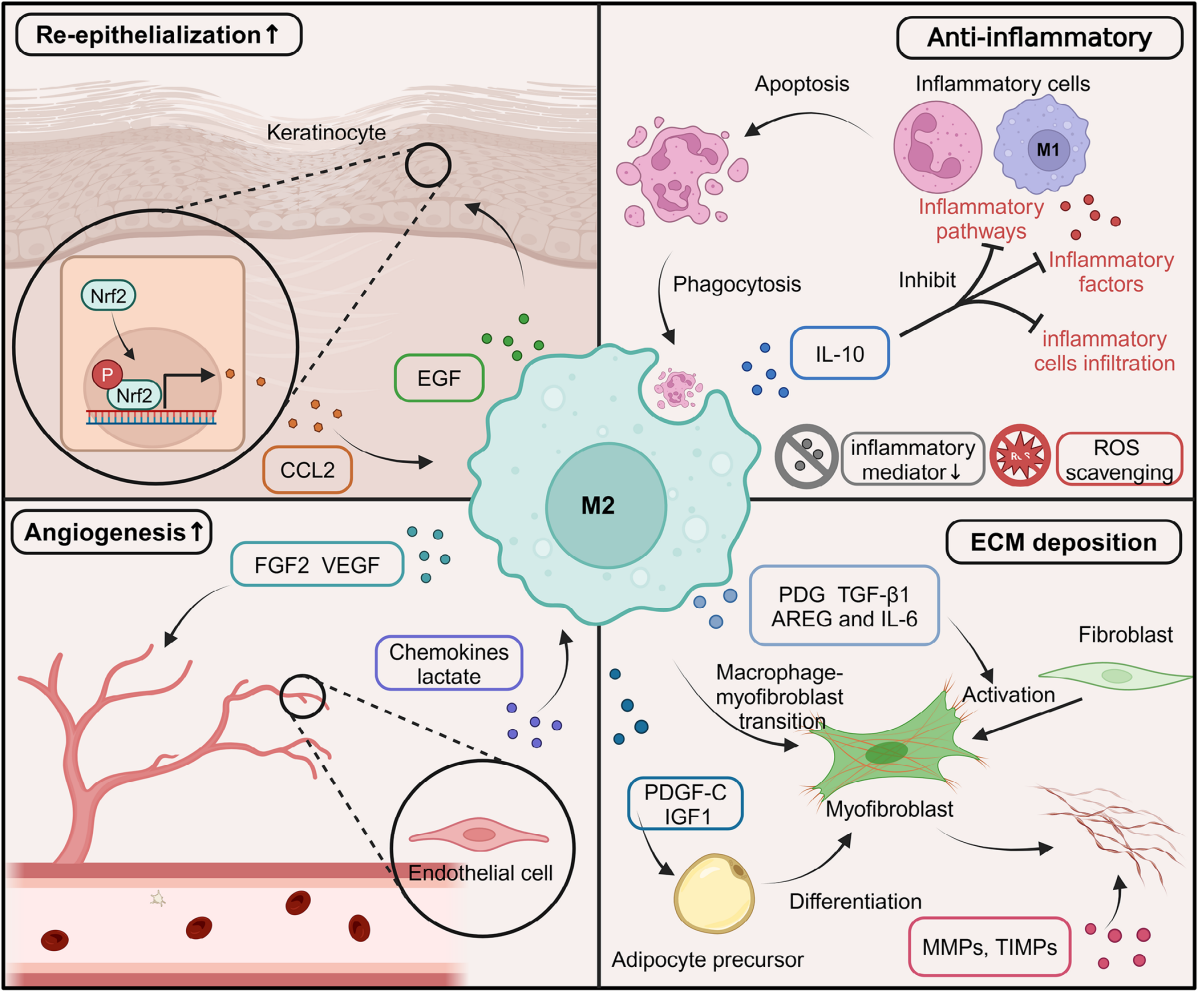
The role of M2‐type macrophages in wound healing. After the inflammatory period, macrophages are polarized into the M2 phenotype, which exerts anti‐inflammatory functions. In addition, the M2‐type‐Macrophages promote skin cell regeneration by releasing a range of cytokines and shaping the immune microenvironment, acting as key immune cells in tissue regeneration, including epidermal regeneration, vascularization, and ECM remodeling mediated by activated fibroblast. AREG, amphiregulin; CCL2, C‐C motif chemokine ligand 2; ECM, extracellular matrix; FGF2, fibroblast growth factor 2; IGF‐1, insulin‐like growth factor 1; IL‐6, interleukin‐6; MMPs, matrix metalloproteinases; MMT, macrophage‐myofibroblast transition; Nrf2, NF‐E2‐related factor 2; PDGF, platelet‐derived growth factor; TGF‐β1, transforming growth factor‐beta1; TIMPs, tissue inhibitor of metalloproteinases; VEGF, vascular endothelial growth factor. Created with BioRender.com.

M2 macrophages modulate the activity of various ECM‐remodeling enzymes, such as MMPs and tissue TIMPs, essential for ECM turnover and remodeling.^378^ During the proliferation and remodeling phase, M2 macrophages produce specific MMPs, such as MMP‐2 and MMP‐9, which facilitate the breakdown of existing ECM components, creating space for the deposition of new ECM and the migration of cells involved in tissue regeneration.^379^, ^380^ However, excessive and uncontrolled MMP activity can lead to excessive ECM degradation and impair tissue repair. To maintain a balance between ECM degradation and deposition, M2 macrophages also produce TIMPs, which are endogenous inhibitors of MMPs. TIMPs bind to and inactivate MMPs, regulating their proteolytic activity and preventing excessive ECM breakdown.^381^, ^382^ The production of specific MMPs and TIMPs by M2 macrophages is tightly controlled and depends on the stage of tissue repair and the microenvironmental cues present. For example, during the early stages of the proliferation and remodeling phase, M2 macrophages may produce higher levels of MMPs to facilitate the initial breakdown of the ECM and create space for new tissue formation.^383^, ^384^ As the tissue repair process progresses, M2 macrophages may shift toward producing higher levels of TIMPs to stabilize the newly formed ECM and promote tissue maturation.^385^ In addition to regulating MMPs and TIMPs, M2 macrophages modulate the activity of other ECM‐remodeling enzymes, such as lysyl oxidases (LOXs) and transglutaminases. LOXs catalyze the cross‐linking of collagen and elastin fibers, increasing the stability and mechanical strength of the ECM. In contrast, transglutaminases catalyze the formation of covalent cross‐links between ECM proteins, further contributing to ECM stabilization and maturation^386^, ^387^, ^388^ (Figure 2).

#### 4.2.4 Macrophage plasticity and phenotypic transitions

**FIGURE 3:**
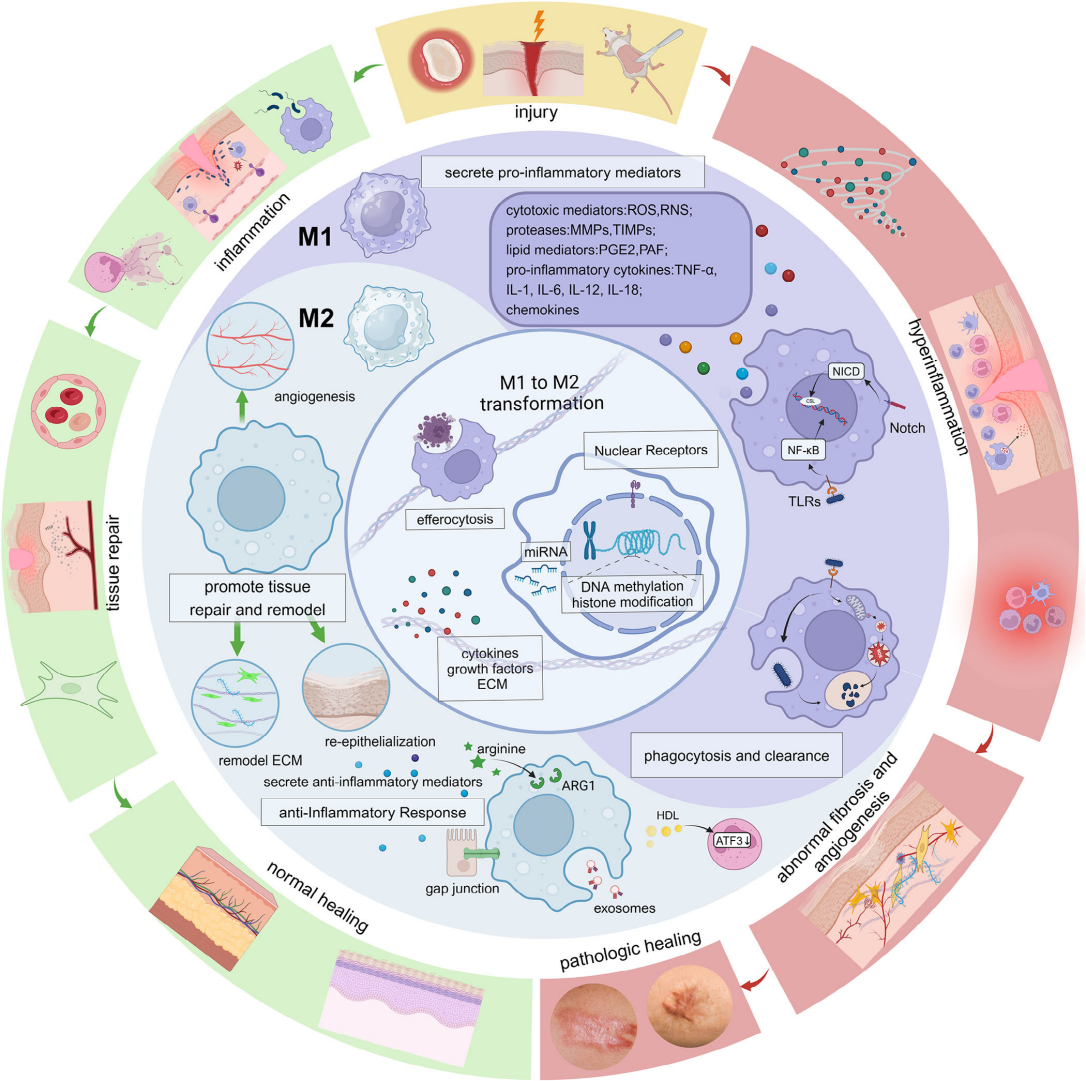
Distinctive roles of M1 and M2 in normal and pathological wound healing. M1 and M2 types of macrophages switch according to a chronological phenotype in the wound and work together concertedly to regulate routine wound healing. Tissue repair is completed as the four overlapping events of hemostasis, inflammation, and remodeling occur. During this process, monocytes can repair the wound by recognizing damage‐associated molecular patterns (DAMPs) and pathogen‐associated molecular patterns (PAMPs). Patterns (PAMPs) and differentiate into M1‐type macrophages stimulated by specific signals from the inflammatory microenvironment, producing cytotoxic mediators (ROS, RNS), proteases (MMP, TIMPS), lipid mediators (PGE2, PAF) proinflammatory factors (TNF‐α, IL‐1, IL‐6 IL‐12, IL‐18) chemokines, phagocytosis and clearance of pathogens and tissue debris. Macrophages then polarize from the M1 to the M2 phenotype, promoting angiogenesis, extracellular matrix (ECM) deposition, and re‐epithelialization with their anti‐inflammatory properties. The M1 and M2 types of macrophages remain in unison and coordination, with excessive M1 macrophage activation leading to tissue damage and chronic inflammation and, conversely, excessive M2 macrophage activation leading to scarring and fibrosis. Created with BioRender.com.

While the M1 and M2 phenotypes represent the extremes of the macrophage activation spectrum, it is crucial to recognize that macrophages exhibit a remarkable degree of plasticity, capable of adopting a wide range of functional states along a continuum. This plasticity allows macrophages to adapt dynamically to the changing microenvironmental cues encountered during tissue repair and regeneration.^389^, ^390^, ^391^ Recent studies have subdivided M2 macrophages into subgroups, including M2a, M2b, M2c, and M2d, based on their upstream activators and downstream gene expression patterns.^103^, ^104^, ^392^ For example, M2a macrophages are activated by IL‐4 and IL‐13, exhibiting increased expression of IL‐10, TGF‐β, and chemokines like CCL17, CCL18, and CCL22. In contrast, M2c macrophages are activated by glucocorticoids, IL‐10, and TGF‐β and exhibit increased transcription of IL‐10, TGF‐β, CCL16, and CCL18.^393^, ^394^, ^395^ This classification highlights macrophages’ complex nature and ability to modify their gene transcription profiles along a continuous spectrum, especially in pathological situations. It is important to note that the M1 and M2 phenotypes represent simplified extremes of a heterogeneous and dynamic functional continuum rather than distinct and mutually exclusive populations. Furthermore, macrophages can undergo phenotypic transitions in response to changing microenvironmental signals, allowing them to adapt their functional programs to the evolving needs of the tissue repair process. For example, M1 macrophages may transition to an M2‐like phenotype during the later stages of tissue repair, facilitating the resolution of inflammation and promoting tissue regeneration^396^, ^397^, ^398^ (Figure 3).

### 4.3 Macrophages in tissue‐specific repair and regeneration

While the general principles of macrophage involvement in tissue repair and regeneration are consistent across various organ systems, some tissue‐specific nuances and mechanisms highlight the versatility and adaptability of these cells. Here, we will briefly discuss the roles of macrophages in the repair and regeneration of selected tissues, including skeletal muscle, liver, heart, and skin.

#### 4.3.1 Skeletal muscle repair and regeneration

In skeletal muscle injury, macrophages are crucial in coordinating the inflammatory response, promoting myogenesis, and facilitating tissue remodeling.^399^, ^400^, ^401^ M1 macrophages are the first to infiltrate the injured muscle, where they phagocytose debris and release proinflammatory cytokines to initiate the repair process.^402^, ^403^ Subsequently, a phenotypic switch occurs, and M2 macrophages become predominant. These anti‐inflammatory macrophages secrete factors like IGF‐1 and TGF‐β that stimulate myoblast proliferation and differentiation.^404^, ^405^, ^406^ M2 macrophages also produce MMPs that degrade the extracellular matrix, allowing myoblast migration and fusion into multinucleated myotubes.^407^ Recent studies have revealed that macrophages can directly interact with muscle stem cells (satellite cells) through cell–cell contacts and paracrine signaling.^408^, ^409^, ^410^ These interactions regulate satellite cell quiescence, activation, proliferation, and differentiation, ensuring proper muscle regeneration. Furthermore, macrophages contribute to the revascularization of the regenerating muscle by producing proangiogenic factors like VEGF and promoting the formation of new blood vessels.^87^, ^411^ This process is essential for delivering nutrients and oxygen to the newly formed muscle fibers.

#### 4.3.2 Liver regeneration

The liver has a remarkable capacity for regeneration, and macrophages play a pivotal role in this process.^412^ Following partial hepatectomy or liver injury, Kupffer cells (liver‐resident macrophages) and infiltrating monocyte‐derived macrophages orchestrate the regenerative response.^413^, ^414^, ^415^ Initially, M1 macrophages promote hepatocyte proliferation by producing TNF‐α and IL‐6. They also phagocytose debris and apoptotic cells, creating space for the regenerating liver tissue.^416^, ^417^, ^418^ As regeneration progresses, M2 macrophages dominate and secrete factors like Wnt proteins, EGF, and MMPs that support hepatocyte proliferation, migration, and matrix remodeling.^419^, ^420^, ^421^ M2 macrophages also produce anti‐inflammatory cytokines like IL‐10 to resolve inflammation and prevent excessive tissue damage.^416^, ^422^ Recent studies have highlighted the importance of macrophage–hepatocyte crosstalk in regulating liver regeneration. Macrophages respond to signals from hepatocytes and modulate their phenotype and function accordingly, creating a feedback loop that fine‐tunes the regenerative process.^423^, ^424^

#### 4.3.3 Cardiac repair and regeneration

While the adult mammalian heart has limited regenerative capacity, macrophages play a crucial role in regulating the inflammatory response and facilitating cardiac repair following myocardial infarction (MI).^425^, ^426^ After MI, M1 macrophages infiltrate the infarcted area and initiate the inflammatory response by producing proinflammatory cytokines and chemokines.^427^ They also phagocytose necrotic cardiomyocytes and debris, preparing the area for subsequent repair. As inflammation resolves, M2 macrophages become predominant and promote angiogenesis, extracellular matrix deposition, and scar formation. They secrete factors like VEGF, TGF‐β, and PDGF that stimulate endothelial cell proliferation, fibroblast activation, and collagen deposition.^428^, ^429^ Interestingly, recent studies have suggested that macrophages may also play a role in cardiac regeneration by modulating the behavior of cardiac progenitor cells and cardiomyocytes.^430^, ^431^ M2 macrophages can secrete factors like oncostatin M and IL‐33 that promote cardiomyocyte proliferation and survival, potentially contributing to new cardiac muscle.^432^, ^433^ However, excessive inflammation and prolonged M1 macrophage activation can lead to adverse cardiac remodeling and heart failure.^434^, ^435^ Therefore, modulating macrophage phenotypes and functions may represent a therapeutic strategy for improving cardiac repair and regeneration.

#### 4.3.4 Skin wound healing

Macrophages are essential for proper skin wound healing, involving inflammation, tissue formation, and remodeling. During the inflammatory phase, M1 macrophages infiltrate the wound site and phagocytose pathogens, debris, and apoptotic cells. They also release proinflammatory cytokines and chemokines to recruit additional immune cells and initiate the repair process.^1^, ^2^ As the inflammatory phase resolves, M2 macrophages become predominant and promote tissue formation and remodeling.^63^ They secrete growth factors like VEGF, TGF‐β, and PDGF that stimulate angiogenesis, keratinocyte migration and proliferation, and extracellular matrix deposition.^436^ M2 macrophages also play a role in wound contraction and scar formation by producing factors that activate fibroblasts and promote collagen deposition.^437^, ^438^, ^439^ Additionally, they secrete anti‐inflammatory cytokines like IL‐10 to resolve inflammation and prevent excessive tissue damage.^440^ Recent studies have highlighted the importance of macrophage‐keratinocyte crosstalk in regulating skin wound healing.^441^, ^442^ Macrophages respond to signals from keratinocytes, modulate their phenotype, and function accordingly, creating a feedback loop that fine‐tunes the repair process.

### 4.4 Macrophage dynamics in chronic wounds

Chronic wounds, characterized by their persistent inflammatory state and impaired healing, pose a significant challenge in clinical settings. Among the various types of chronic wounds, diabetic wounds stand out as a significant concern due to their increasing prevalence and the unique microenvironment that hinders the healing process.^443^ In recent years, the role of macrophages in the pathogenesis and resolution of diabetic wounds has garnered significant attention.^62^, ^444^ This section delves into the complex interplay between macrophages and the diabetic wound microenvironment, highlighting the mechanisms that influence macrophage phenotype and function and exploring potential therapeutic strategies targeting these interactions.

Diabetic wounds, particularly diabetic foot ulcers, are a common and severe complication of diabetes mellitus. The global prevalence of diabetic foot ulcers is estimated to be 6.3%, with a lifetime incidence of up to 25% among diabetic patients.^445^, ^446^ These wounds are characterized by a prolonged inflammatory phase, impaired angiogenesis, and delayed re‐epithelialization, leading to a chronic nonhealing state. The unique microenvironment of diabetic wounds, shaped by hyperglycemia, oxidative stress, and the accumulation of advanced glycation end products (AGEs), significantly influences the behavior and function of macrophages, which are critical players in the wound healing process.^447^, ^448^

#### 4.4.1 The influence of the diabetic wound microenvironment on macrophage phenotype

In diabetic wounds, the local microenvironment is skewed toward factors promoting a persistent M1 phenotype, leading to chronic inflammation and impaired healing. High glucose levels, a hallmark of diabetes, have been shown to directly influence macrophage polarization.^449^ In vitro, studies have demonstrated that exposure to high glucose concentrations enhances the expression of proinflammatory cytokines, such as TNF‐α and IL‐1β, in macrophages while suppressing the expression of anti‐inflammatory markers, such as IL‐10 and ARG‐1.^450^, ^451^ This shift toward an M1 phenotype is mediated through the activation of signaling pathways, including NF‐κB and MAPK, which are known to regulate inflammatory responses.^452^, ^453^, ^454^

Oxidative stress, another key feature of the diabetic wound microenvironment, also plays a crucial role in modulating macrophage phenotype. ROS, such as superoxide and hydrogen peroxide, are elevated in diabetic wounds due to hyperglycemia‐induced mitochondrial dysfunction and the activation of NADPH oxidase.^455^, ^456^ Excessive ROS levels contribute to the persistent activation of proinflammatory signaling cascades, such as the NF‐κB pathway, in macrophages.^452^, ^457^ Moreover, ROS can directly damage macrophages, impairing their phagocytic function and ability to transition toward an M2 phenotype, which is essential for wound resolution.^54^, ^458^

AGEs, formed by the nonenzymatic glycation of proteins and lipids under hyperglycemic conditions, accumulate in the diabetic wound bed and contribute to impaired healing.^459^, ^460^ AGEs interact with their receptor (RAGE) on macrophages, triggering proinflammatory signaling pathways, such as NF‐κB and MAPK, and inducing the production of ROS and proinflammatory cytokines.^461^, ^462^, ^463^ The AGE–RAGE interaction also impairs macrophage efferocytosis, a process critical for the clearance of apoptotic cells and the resolution of inflammation.^464^ Consequently, the accumulation of AGEs in diabetic wounds perpetuates a state of chronic inflammation and hinders the transition of macrophages toward an M2 phenotype.

#### 4.4.2 Mechanisms underlying the effects of glucose, ROS, and AGEs on macrophages

The mechanisms by which high glucose, ROS, and AGEs influence macrophage function in diabetic wounds are complex and multifaceted. High glucose levels can directly alter macrophage metabolism, shifting it toward a more glycolytic phenotype associated with the M1 polarization state.^96^, ^465^ This metabolic reprogramming is mediated through the activation of HIF‐1α and the upregulation of glycolytic enzymes, such as hexokinase and pyruvate kinase^466^, ^467^, ^468^ The increased glycolytic flux in macrophages promotes the production of proinflammatory cytokines and impairs their ability to engage in oxidative phosphorylation, which is necessary for the M2 phenotype.

Oxidative stress, driven by elevated ROS levels, contributes to the persistent activation of redox‐sensitive transcription factors, such as NF‐κB and AP‐1, in macrophages.^469^, ^470^ These transcription factors regulate the expression of proinflammatory genes, including TNF‐α, IL‐1β, and IL‐6, perpetuating the inflammatory response in diabetic wounds. ROS can also directly damage macrophages by inducing lipid peroxidation, protein carbonylation, and DNA damage, impairing their function and survival.^471^, ^472^, ^473^ Furthermore, ROS‐mediated oxidative modifications of proteins can generate new AGEs, amplifying the AGE–RAGE signaling loop and exacerbating inflammation.^474^

AGEs interact with RAGE on macrophages, triggering a cascade of signaling events that promote the M1 phenotype.^475^, ^476^ The AGE–RAGE interaction activates NF‐κB and MAPK pathways, leading to the transcription of proinflammatory genes and the production of ROS.^477^ Additionally, AGEs can induce epigenetic modifications in macrophages, such as histone acetylation and DNA methylation, which regulate gene expression in inflammation and wound healing.^478^, ^479^ For example, AGEs have been shown to increase the acetylation of histone H3 at the promoter regions of proinflammatory genes, such as TNF‐α and IL‐1β, enhancing their transcription.^480^ AGEs also impair macrophage efferocytosis by downregulating the expression of efferocytosis receptors, such as MerTK and CD36, and by inducing the production of “don't eat me” signals, such as CD47, on apoptotic cells.^481^, ^482^

#### 4.4.3 Targeting macrophage‐microenvironment interactions for diabetic wound treatment

Understanding the complex interactions between macrophages and the diabetic wound microenvironment provides valuable insights for developing targeted therapeutic strategies. One promising approach is modulating the ROS levels in the wound bed. Antioxidants, such as N‐acetylcysteine and vitamin E, have reduced oxidative stress and improved wound healing in diabetic animal models.^483^, ^484^, ^485^ These antioxidants scavenge ROS, attenuate the activation of proinflammatory signaling pathways, and promote the polarization of macrophages toward an M2 phenotype. Clinical studies have also demonstrated the potential of topical antioxidant application in improving diabetic wound healing, highlighting the translational relevance of targeting ROS.^486^, ^487^

Another strategy is inhibiting AGE formation and accumulation of AGEs in the wound bed. Pharmacological agents, such as aminoguanidine and pyridoxamine, have been shown to reduce AGE formation and improve wound healing in diabetic animal models.^488^, ^489^ These compounds trap reactive carbonyl intermediates and prevent their condensation with proteins to form AGEs. Additionally, targeting the AGE–RAGE signaling axis using RAGE antagonists or soluble RAGE (sRAGE) has shown promise in preclinical studies.^490^ sRAGE acts as a decoy receptor, sequestering AGEs and preventing their interaction with cell surface RAGE, thus attenuating proinflammatory signaling in macrophages.^491^, ^492^

Modulating macrophage metabolism is another potential therapeutic approach. Compounds that promote oxidative phosphorylation and mitochondrial biogenesis, such as resveratrol and metformin, have been shown to skew macrophages toward an M2 phenotype and improve wound healing in diabetic animal models.^493^, ^494^, ^495^, ^496^ These compounds activate AMPK to promote the expression of anti‐inflammatory genes and suppress glycolysis. Clinical trials investigating the effects of metformin on diabetic wound healing have shown promising results, with improved wound closure rates and reduced inflammation.^497^

In conclusion, macrophages are versatile and dynamic cells that play pivotal roles throughout the various tissue repair and regeneration phases. Their remarkable plasticity and ability to adapt to changing microenvironmental cues allow them to orchestrate various processes, from initiating inflammation and pathogen clearance to promoting angiogenesis, extracellular matrix remodeling, and tissue regeneration. As our understanding of macrophage biology continues to deepen, these cells hold great promise as therapeutic targets for enhancing tissue repair and regenerative processes in various pathological conditions.

## 5 HARNESSING MACROPHAGES: TARGETING PATHWAYS FOR TISSUE REPAIR AND REGENERATION

**FIGURE 4:**
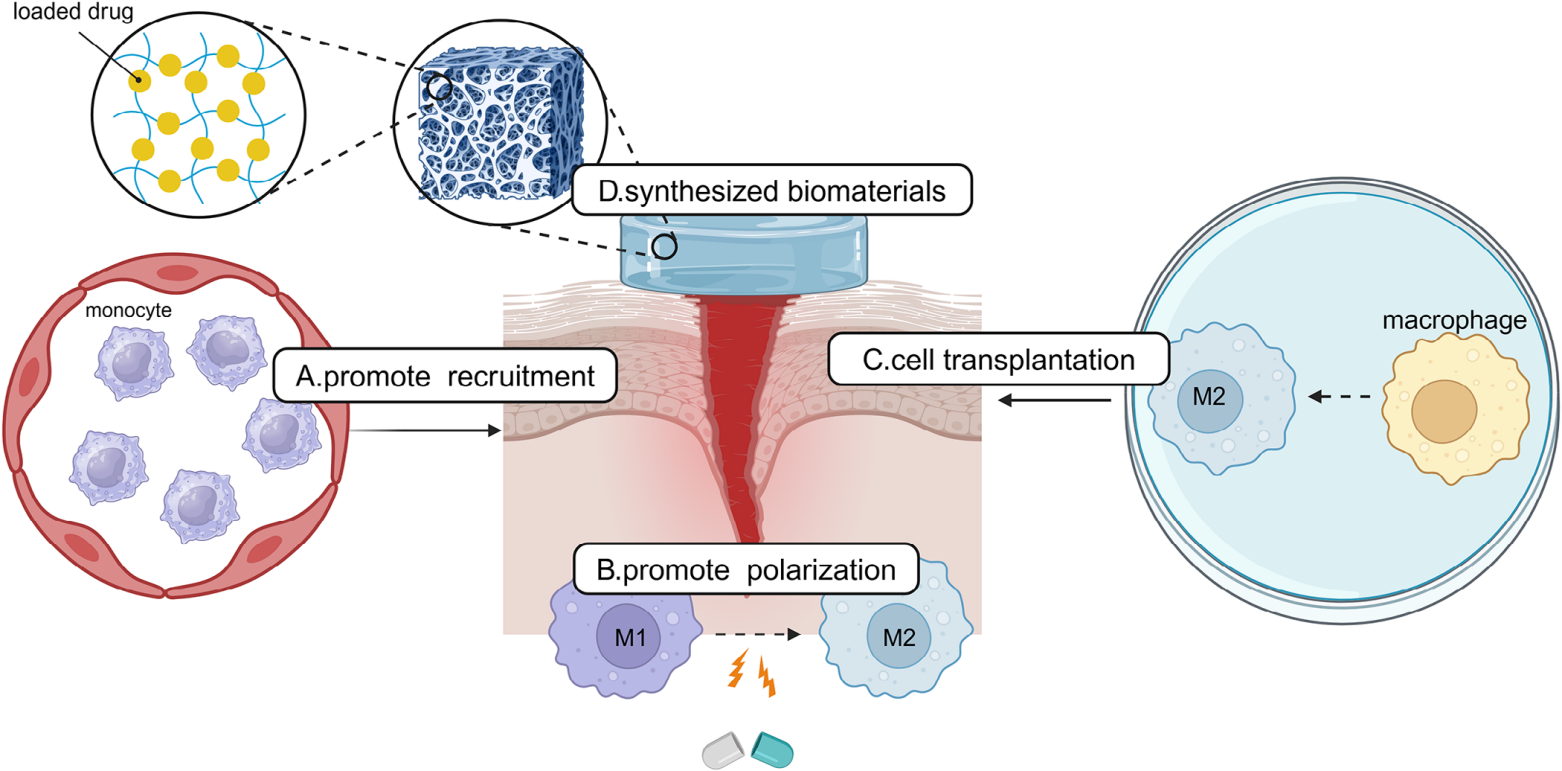
Therapeutic strategies for wound healing targeting macrophages. There are currently the following potential therapeutic strategies for wound healing targeting macrophages: (A) Promote monocyte recruitment to increase the number of macrophages in the wound. (B) Promote the timely polarization of proinflammatory M1 macrophages in the wound into an anti‐inflammatory and prorepair M2 phenotype. (C) Macrophages were induced to express the M2 phenotype in vitro, and the macrophages promoting repair were transplanted into the wound tissue site. (D) The use of biosynthetic materials to precisely regulate the surface transformation of macrophages, such as hydrogels, can promote wound repair through different substances synthesized by hydrogels or drugs supported by them. Created with BioRender.com.

Macrophages, the versatile cells of the innate immune system, have emerged as pivotal players in the intricate tissue repair and regeneration process. Recent advancements in regenerative medicine and molecular biology have shed light on the critical role of macrophages in promoting the regeneration of various tissues, including the heart, liver, kidney, muscle, and nerves. The ability of macrophages to adopt diverse phenotypes in response to microenvironmental cues has made them attractive therapeutic targets for enhancing tissue repair and regeneration. This section explores the current strategies for harnessing macrophages to promote tissue repair and regeneration, focusing on the latest research findings from the past decade. We discuss targeting specific pathways, such as the CSF‐1/CSF‐1R signaling pathway, and the modulation of macrophage function through signaling pathways and transcription factors. Additionally, we highlight the potential of relay transfer and cell transplantation of macrophages and biomaterial‐based strategies for precise regulation of macrophage polarization phenotypes (Figure 4).

### 5.1 Targeting the CSF‐1/CSF‐1R signaling pathway

The macrophage CSF‐1 and its receptor (CSF‐1R) signaling pathway play a crucial role in the maturation and transformation of TrMΦ, making it an attractive target for therapeutic^498^, ^499^, ^500^, ^501^ interventions. Stutchfield et al.^502^ demonstrated that the administration of CSF1‐Fc, an exogenous form of CSF‐1, enhanced the recruitment and conversion of monocytes into protective hepatic macrophages, facilitating liver recovery after acute injury and partial hepatectomy. Furthermore, a systematic review and meta‐analysis by Wei et al.^503^ provided robust evidence supporting the efficacy and safety of CSF in accelerating wound healing. In addition to CSF‐related approaches, targeting chemokine receptors on monocytes and macrophages has shown promise in altering their migration patterns and impacting their function in tissue repair.^169^, ^504^ Promoting the survival of M2 macrophages, which play an essential role in tissue repair and angiogenesis, represents another promising strategy for enhancing tissue healing.

### 5.2 Modulating macrophage function through signaling pathways and transcription factors

Manipulating macrophage function by targeting specific signaling pathways and transcription factors has emerged as a promising therapeutic strategy for promoting tissue repair and regeneration. The TLR9 signaling pathway has been found to encourage macrophage M2 polarization in various models.^505^ Activation of the TLR9 pathway, such as with the agonist cobitolimod, induces a prohealing phenotype in macrophages, enhancing macrophage‐mediated tissue healing in conditions like ulcerative colitis.^506^ Transcription factors, including NF‐κB and MAPK, play critical roles in macrophage activation and polarization.^507^, ^508^ Inhibiting these signaling pathways has shown promising results in promoting M2 polarization and reducing inflammation. Puerarin, a compound from traditional Chinese medicine, has demonstrated the ability to inhibit NF‐κB and MAPK pathways, leading to decreased production of inflammatory cytokines and promotion of M2 polarization in macrophages.^99^, ^509^, ^510^ Noncoding RNAs, particularly miRNAs, have also emerged as important macrophage polarization and activation regulators. Targeting specific noncoding RNAs provides a means to modulate macrophage function and influence tissue repair processes.^511^, ^512^ For instance, inhibiting the pro‐M1 polarization molecule CRMP2 through small interfering RNA has demonstrated reduced local inflammation and fibrosis following MI.^513^

### 5.3 Relay transfer and cell transplantation of macrophages

Targeting specific subpopulations of macrophages through relay transfer and cell transplantation has shown great potential for clinical treatment aimed at tissue repair and regeneration.^514^ Lopes et al.^515^ demonstrated the efficacy of modifying macrophages to express the M2 phenotype in vitro and subsequently transferring these M2 macrophages to a colitis mouse model, reducing inflammation and pathological damage. Similarly, Zheng et al.^516^ stimulated macrophages to adopt the M2 phenotype using IL‐4 and IL‐13. They transplanted these M2 macrophages into a streptozotocin‐induced diabetic mouse model, significantly reducing damage in the islets and kidneys. These studies highlight the potential of relay transfer and polarized macrophage cell transplantation to promote tissue repair and regeneration. However, further research is needed to fully understand the mechanisms of macrophage polarization and develop more precise and effective strategies for modulating macrophage function in vivo.^517^ This includes identifying specific markers and signaling pathways that regulate macrophage polarization and investigating methods to optimize the survival and functionality of transplanted macrophages.

### 5.4 Biomaterial‐based strategies for precise regulation of macrophage polarization

Recent advancements in biomaterials have enabled precise regulation of macrophage polarization phenotypes, specifically M1/M2, leading to enhanced tissue regeneration and accelerated wound healing.^518^, ^519^, ^520^, ^521^, ^522^, ^523^ Hydrogel‐based constructs, known for their biocompatibility, tunable physical properties, and drug‐delivery capabilities, have emerged as valuable tools for tissue repair.^524^, ^525^, ^526^ Huang et al.^527^ developed curcumin‐based metal‐organic framework hydrogels that effectively downregulated M1 macrophage‐related gene expression while upregulating anti‐inflammatory gene expression, promoting the polarization of macrophages toward the M2 phenotype and facilitating the regeneration of blood vessels and nerves in chronic wounds. Henn et al.^528^ investigated xenotransplantation‐mediated activation of Trem2+ macrophages, which promoted re‐epithelialization and angiogenesis through growth factor secretion and contributed to collagen remodeling by secreting MMPs.^378^ By designing a soft pullulan‐collagen hydrogel, delivery of Trem2+ macrophages obtained after vitamin D3 treatment to the wound bed showed great potential for clinical translation. Biomaterials offer a platform for precise modulation of macrophage function and polarization through tailored design and incorporation of specific cues, such as drug delivery systems or bioactive molecules.^529^, ^530^, ^531^, ^532^ Further research is needed to optimize the design and functionality of biomaterials to achieve better control over macrophage polarization and ultimately improve clinical outcomes in tissue repair and regeneration (Table 3).

**TABLE 3:** Currently, biomaterial strategies for targeting macrophages and their functions in wound healing.

Hydrogel composition		Bioactive molecules	Effect of hydrogel dressings	References
Natural hydrogel	Agarose	Carrageenan	Promote macrophage to enter the proinflammatory M1 type	^533^
Natural hydrogel	Hyaluronic acid	Paeoniflorin	Modulating the phenotype of macrophages from M1 to M2	^534^
		H_2_S	Promoting macrophage polarization toward M2 phenotype and inhibiting M1 polarization	^535^
		MiR‐223	Increased the level of macrophage infiltration but also effectively mediated the local polarization of macrophages toward the M2 phenotype	^536^
		Tetramethylpyrazine	Activation of M2 macrophage function via STAT signaling pathway	^537^
Natural hydrogel	Gelatin	Snail glycosaminoglycan	By sequestrating proinflammatory cytokines and downregulating their expression by inhibiting the NF‐ĸB signaling pathway, macrophages were promoted to polarization toward the M2 phenotype.	^538^, ^539^
Natural hydrogel	Chitosan	Collagen type I	Promote the production of anti‐inflammatory cytokines in macrophages and selectively reduce the production of proinflammatory cytokines. The polarization of macrophages toward the M2 subgroup was directly induced, and the M1 polarization of wound bed macrophages was reversed.	^540^
		PGE2	Inhibited the infiltration of inflammatory cells and secretion of proinflammatory cytokines and promoted the polarization of M2 macrophages at the injured site	^541^
		Phosphocreatine	Inhibits the expression of proinflammatory cytokine chemokines in macrophages stimulated by INF	^542^
		Bone marrow mesenchymal stem cell‐derived exosome	Promotes the phenotype transition from M1 to anti‐inflammatory M2 and inhibits the release of various proinflammatory cytokines	^543^
		Melanin composite nanoparticles	Promoted the polarization of M1 macrophage to M2 macrophage and activated autophagy of M2 macrophages	^544^
Natural hydrogel	Chitosan/agarose	Vitamin C	Reduces the ability of macrophages to produce MMP1 and MMP2	^545^
Natural hydrogel	Agarose‐grafting‐hyaluronan scaffold	Agarose‐grafting‐hyaluronan scaffold	Accelerate cell proliferation and stimulate secretion of TNF‐α for macrophages.	^546^
Natural hydrogel	Curdlan/agarose	Vitamin C and hydrocortisone	Significantly decrease release of MMP‐2 by human macrophages	^547^
	Curdlan/chitosan			
Synthetic hydrogel	Polyacrylamide	Glucose oxidase	Induce the transformation of macrophages to M2 phenotype, accelerate the transformation of wound microenvironment to remodeling state, and then accelerate angiogenesis and neurogenesis	^548^
Synthetic hydrogel	Polyvinyl alcohol	ROS‐responsive linker, mupirocin, and GM‐CSF	Reduces inflammation, reduces the secretion of various proinflammatory cytokines, increases the percentage of M2‐type macrophages, and triggers the production of new blood vessels and collagen around the wound	^549^
		Cholinium salicylate, cholinium gallate, cholinium vanillate and cholinium caffeate	The decrease in LPS‐induced NO production indicated that the material had anti‐inflammatory activity	^550^
		Aloe gel extract	Downregulate the expression of the proinflammatory gene, IL‐6 and iNOS, and significantly inhibit the production of reactive oxygen species	^551^
Synthetic hydrogel	Methyl methacrylate	Glycyrrhizic acid, Zn2+	By regulating the ratio of M1/M2 macrophages rather than ablating macrophages, the three stages of diabetic wound repair were accelerated.	^552^
Synthetic hydrogel	polyvinyl alcohol/polyvinylidene fluoride	Piezoelectric response	Regulate macrophage phenotype from the M1 subtype to the M2 subtype, and the expression level of inflammatory factors is reduced through the AKT and ERK1/2 signaling pathways.	^553^
Composite hydrogel	Chitosan/polyvinyl alcohol	Ursolic acid	Reduce the M1 phenotype transformation of macrophages stimulated by lipopolysaccharide, effectively restore the M2 polarization of macrophages, and shorten the inflammatory period	^554^
Composite hydrogel	Gelatin methacrylate and silk fibroin glycidyl methacrylate	Platelet‐derived extracellular vesicles, resveratrol	Inhibiting the expression of proinflammatory factors TNF‐α and iNOS in macrophages, increasing the expression of anti‐inflammatory factors TGF‐β1 and Arg‐1, promoting angiogenesis, and accelerating wound healing	^555^
Composite hydrogel	Polylactic acid/bletilla striata polysaccharide/rosmarinic acid	Rosmarinic acid, bletilla striata polysaccharide	Transform M1 macrophages into M2 macrophages, reduce the release of inflammatory factors, and promote effective wound healing.	^556^
Composite hydrogel	Chitosan/polyvinyl alcohol/gelatin	Chitosan/polyvinyl alcohol/gelatin	Macrophages moderately induced M1‐type polarization, which made them have specific phagocytic potential, and induced M2‐type polarization in the late healing period	^557^
Composite hydrogel	Polyvinyl alcohol/xanthan gum/hypromellose/sodium carboxymethyl	Silver nanoparticles	Reduce the production of H_2_O_2_‐mediated inflammatory response in macrophages	^558^

Abbreviations: ARG, arginine; INF, interferon; iNOS, inducible nitric oxide synthase; LPS, lipopolysaccharides; MiR, microRNA; MMP, matrix metalloproteinase; PGE2, prostaglandin E2; STAT, signal transducer and activator of transcription; TGF, transforming growth factor; TNF, tumor necrosis factor.

### 5.5 Therapeutic potential and clinical applications

The therapeutic potential of macrophages in cell‐based therapies has been demonstrated in various clinical trials and preclinical studies.^522^, ^559^, ^560^ Macrophages have outperformed stem cells in specific target diseases, showcasing their outstanding regenerative capacity.^561^ Conditions such as kidney disease, stroke, arterial disease, and cancer have been targeted using macrophage‐based therapies. Genetic modification of macrophages, such as the development of chimeric antigen receptor‐macrophages (CAR‐M), has further expanded the potential of genetically engineered macrophages for cell therapy.^562^, ^563^, ^564^ The use of induced pluripotent stem cell (iPSC)‐derived macrophages, macrophages loaded with nanoparticles, ex vivo polarization and adoptive transfer of macrophages, and surface‐anchoring engineering of macrophages have also shown promising results in preclinical studies.^565^, ^566^, ^567^ The therapeutic applications of macrophage CSF‐1 have been explored in various contexts, including tissue repair after ischemia in the kidney and heart, promotion of angiogenesis, and elimination of amyloid deposits in the brain.^568^, ^569^, ^570^ CSF‐1 has been shown to promote a resident‐type macrophage phenotype, making it a potential treatment for tissue repair (Table 4).

**TABLE 4:** Clinical study on regulating macrophages to improve wound healing.

Treatment measure	Applied disease	Effect on macrophages	Therapeutic effect	References
ON101	DFUs	Decreasing inflammatory M1 macrophage activity and enriching M2 macrophage populations	ON101 showed significant efficacy in diabetic ulcers lasting 6 months or larger than 5cm2	^522^
rhGM‐CSF	Deep second‐degree burn wound	Stimulate macrophages’ maturation and rapid recruitment, save damaged macrophages, and accelerate wound repair	The wound healing is accelerated, the formation of capillaries is accelerated, and the scar after healing is reduced.	^571^
	Deep second‐degree burns of infants		It is beneficial for controlling infection, accelerating scab dissolution, and inhibiting pathological scarring formation.	^572^
	Third‐degree frostbite wound		Improves wound healing and inflammation levels and reduces the risk of infection	^573^
NPWT	DFUs to be treated with STSG	Macrophages were polarized from M1 to M2	Increased survival of skin grafts	^574^
TR‐987 0.1% active gel	Wound after laser resurfacing	Mildly increases the proinflammatory phenotype and initiates the wound repair cascade	Skin quality after healing (elastosis and wrinkling) is significantly improved.	^575^
YaSP	DFUs	Inhibition of nitric oxide production in M1 macrophages	Accelerated the speed of diabetic wound healing	^576^
Alveofact	Human suction blister wound	The number of M1 macrophages in the wound was decreased, and the secretion of inflammatory cytokines was decreased.	The speed of wound re‐epithelialization and wound healing were accelerated.	^577^
Expressive writing	Punch biopsy wound	Langerhans cell infiltration and duration increased, and macrophage M1 polarization decreased during healing.	Wound re‐epithelialization and healing were accelerated.	^578^
Cobitolimod	Ulcerative colitis	Macrophages are stimulated to secrete IL‐10 by TRL9.	Improve the dysregulation of intestinal cytokines and excessive inflammation	^506^
EGCG	Skin scar	Macrophage M2 polarization increased.	Skin scar elasticity increases, hydration increases, and blood vessel density decreases.	^579^, ^580^
MALP‐2	Punch biopsy wound	MALP‐2 activates macrophages to secrete significant growth factors for wound healing through TLR‐2 and TLR‐6.	The induced wound local inflammation subsided 48h later.	^581^

Abbreviations: DFUs, diabetic foot ulcers; EGCG, epigallocatechin‐3‐gallate; EMD, enamel matrix protein derivative, NPWT, negative pressure wound therapy; rhGM‐CSF, recombinant human granulocyte‐macrophage colony‐stimulating factor; STSG, split‐thickness skin graft; YaSP, Ya‐Samarn‐Phlae.

In summary, macrophages play a crucial role in tissue repair, regeneration, and fibrosis, making them attractive targets for therapeutic interventions. The ability to harness macrophages through various strategies, such as targeting specific signaling pathways, modulating macrophage function, relay transfer and cell transplantation, and biomaterial‐based approaches, holds great promise for enhancing tissue repair and regeneration. Future research should further elucidate the mechanisms that instruct macrophages to adopt specific phenotypes and identify novel targets for therapeutic modulation. The development of more precise and effective strategies for modulating macrophage function in vivo and optimizing the survival and functionality of transplanted macrophages will be crucial for translating these findings into clinical applications. Additionally, the potential of genetically engineered macrophages, such as CAR‐M, and using iPSC‐derived macrophages warrant further exploration. Combining macrophage‐based therapies with other therapeutic modalities, such as biomaterials and drug delivery systems, may also provide synergistic effects and improve clinical outcomes.

## 6 CONCLUSION AND PROSPECTS

Macrophages are remarkably plastic cells that play pivotal roles in tissue homeostasis, inflammation, repair, and regeneration. The past decade has witnessed significant advances in understanding the molecular mechanisms governing macrophage plasticity and their functional implications in health and disease. While providing a helpful framework, the traditional M1/M2 classification system has been challenged by the emergence of a spectrum of activation states revealed by single‐cell technologies. The complex interplay between tissue‐specific factors, ontogeny, and microenvironmental cues shapes macrophages’ transcriptional and epigenetic landscape, giving rise to a diverse array of functional phenotypes. The signaling pathways orchestrating macrophage polarization have been extensively studied, with TLRs, STAT proteins, nuclear receptors, and miRNAs emerging as key regulators. The integration of these signaling cascades, along with metabolic reprogramming and epigenetic modifications, fine‐tunes macrophage responses to various stimuli. Notably, the crosstalk between these pathways and the influence of the tissue microenvironment on macrophage plasticity has been increasingly recognized. The discovery of TrMΦ with distinct ontogenies and the concept of trained immunity have further expanded our understanding of macrophage heterogeneity and their capacity for long‐term functional reprogramming. The functional significance of macrophage plasticity is exemplified by their roles in tissue repair and regeneration. Macrophages orchestrate the inflammatory response, clear cellular debris, and promote angiogenesis, extracellular matrix remodeling, and tissue regeneration. The dynamic transition from proinflammatory to proresolving phenotypes is crucial for successfully executing the repair process. Dysregulation of macrophage function contributes to impaired wound healing, fibrosis, and chronic inflammation, underscoring the therapeutic potential of targeting macrophage polarization in various pathological conditions.

Despite the significant progress in understanding macrophage plasticity, several challenges and opportunities remain. The complex heterogeneity of macrophage phenotypes in vivo and their functional implications in specific tissue contexts warrant further investigation. Developing more sophisticated computational tools and spatial transcriptomics approaches will enable a more comprehensive analysis of macrophage diversity and its interactions with other cells in the tissue microenvironment. Moreover, the mechanisms underlying the crosstalk between signaling pathways and the long‐term epigenetic reprogramming of macrophages in response to environmental challenges require further elucidation. Translating the knowledge of macrophage plasticity into clinical applications is a significant challenge and opportunity. Targeting specific signaling pathways or transcription factors to modulate macrophage function holds promise for treating inflammatory diseases, fibrotic disorders, and impaired wound healing. However, developing targeted therapies that selectively modulate macrophage polarization while minimizing off‐target effects remains a significant hurdle. Nanoparticle‐based drug delivery systems and engineered exosomes have shown potential in delivering therapeutic agents specifically to macrophages, but their clinical translation requires further optimization and safety evaluation. The field of macrophage‐based cell therapies is rapidly evolving, with strategies such as adoptive transfer of ex vivo polarized macrophages, genetic engineering of macrophages, and the use of iPSC‐derived macrophages showing promising results in preclinical studies. However, these engineered macrophages’ long‐term survival, functionality, and safety in vivo need to be carefully assessed. Combining macrophage‐based therapies with other therapeutic modalities, such as biomaterials, growth factors, and immunomodulatory agents, may provide synergistic effects and improve clinical outcomes.

In conclusion, macrophage plasticity is a fundamental property that underlies their diverse functions in tissue homeostasis, inflammation, repair, and regeneration. The past decade has witnessed a paradigm shift in understanding macrophage heterogeneity and the molecular mechanisms governing their polarization. Integrating single‐cell technologies, spatial transcriptomics, and computational approaches has unveiled the complex landscape of macrophage activation states and their functional implications. Targeting macrophage plasticity holds immense therapeutic potential for various diseases, from inflammatory disorders to tissue regeneration and cancer. However, translating these findings into clinical applications requires a deeper understanding of the context‐dependent roles of macrophages, the development of more precise and effective strategies for modulating their function, and rigorous safety and efficacy evaluations. As the field of macrophage biology continues to evolve, interdisciplinary collaborations between immunologists, bioengineers, and clinicians will be crucial in harnessing the power of these versatile cells to benefit human health. With the rapid pace of scientific discoveries and technological advancements, the coming years promise to be an exciting era for macrophage research, with the potential to revolutionize our approach to treating diseases and promoting tissue regeneration.

## AUTHOR CONTRIBUTIONS

Lingfeng Yan and Jue Wang wrote the manuscript and drew the figures. Xin Cai helped design the tables. Yih‐Cherng Liou, Han‐Ming Shen, and Jianlei Hao helped design the manuscript structure and write the manuscript. Canhua Huang, Gaoxing Luo, and Weifeng He evaluated and reviewed the manuscript structure, ideas and science. All authors have read and approved the final manuscript.

## CONFLICT OF INTEREST STATEMENT

Author Canhua Huang and Yih‐Cherng Liou are Editorial board members of Medcomm. Author Canhua Huang and Yih‐Cherng Liou were not involved in the journal's review of or decisions related to this manuscript. The other authors declared no conflict of interest.

## ETHICS STATEMENT

No ethical approval was required for this study.

## Data Availability

Data availability is not applicable to this article as no new data were created or analyzed in this study.
